# Preparation, Stimulus–Response Mechanisms and Applications of Micro/Nanorobots

**DOI:** 10.3390/mi14122253

**Published:** 2023-12-17

**Authors:** Tao He, Yonghui Yang, Xue-Bo Chen

**Affiliations:** School of Electronic and Information Engineering, University of Science and Technology Liaoning, Anshan 114051, China; ht18191863591@163.com (T.H.); yangyh2636688@163.com (Y.Y.)

**Keywords:** microrobot, preparation methods, stimulus–response mechanisms, applications, swarm

## Abstract

Micro- and nanorobots are highly intelligent and efficient. They can perform various complex tasks as per the external stimuli. These robots can adapt to the required functional form, depending on the different stimuli, thus being able to meet the requirements of various application scenarios. So far, microrobots have been widely used in the fields of targeted therapy, drug delivery, tissue engineering, environmental remediation and so on. Although microbots are promising in some fields, few reviews have yet focused on them. It is therefore necessary to outline the current status of these microbots’ development to provide some new insights into the further evolution of this field. This paper critically assesses the research progress of microbots with respect to their preparation methods, stimulus–response mechanisms and applications. It highlights the suitability of different preparation methods and stimulus types, while outlining the challenges experienced by microbots. Viable solutions are also proposed for the promotion of their practical use.

## 1. Introduction

Through a lengthy process of evolution, nature has formed biological machines that possess the ability to carry out intricate tasks with accuracy in various fields, thereby constituting intricate biological systems. Taking inspiration from these natural biological machines, humans have dedicated significant amounts of time and resources towards creating man-made micro-machines. Microrobots represent a crucial advancement in the production of operational micromachines. Microrobots are tiny devices that can convert natural energy into motion or force needed for motion. However, when particles move in low Reynolds number fluids, they experience significant viscous drag. Due to the small size of the micromotor, particles usually move in random Brownian motion, making it challenging to direct their movement in the fluid. Therefore, novel nanomotors and driving mechanisms need to be developed. So far, different fabrication methods have been used to prepare microrobots with the same structure, and then the particles can be driven by various external stimuli. However, these methods have some limitations in terms of scalability and precision.

The purpose of making microbots is to create effective gadgets that can move. So, prior to detailing the particle preparation procedures, we first introduce the different actuation mechanisms used to propel the microbots. Zhou et al. [1] reported on different stimulus–response mechanisms used to propel microbots. Microbots can move through chemical reactions or in the presence of applied fields like acoustic fields [2,3,4,5,6,7], magnetic fields [8,9,10,11,12,13,14,15], light [16,17,18,19,20,21,22,23,24,25,26,27,28] and electric fields. Some researchers have proposed the creation of biohybrid micromotors using biomaterials that are found in nature [29,30,31,32,33,34,35].

Among the various propulsion mechanisms, the use of chemical fuels to power micromotors is of most interest to us. In the presence of added chemical fuels, microbots achieve motion by various mechanisms such as interfacial tensor gradients [36,37], autoelectrophoresis [38,39,40,41,42,43,44,45], self-diffusion electrophoresis [46,47] and bubble recoil [36,48,49,50,51]. The interfacial tensor gradient along the interface causes an unbalanced distribution of forces resulting in flow, also known as the Marangoni effect. Autoelectrophoresis has been widely used to design self-powered micromotors. In the mechanism of autoelectrophoresis, particles achieve motion by means of a local field or concentration difference that they generate themselves. Microrobots that achieve motion by self-diffusion electrophoresis are usually bimetallic Janus particles and nanowires, for example, Pt/Au bimetallic particles. The oxidation and decomposition of H_2_O_2_ takes place at both ends of the particles, and the flow of electrons inside the particles is accompanied by the migration of protons outside the particles, causing the particles to move in the opposite direction. In the case of self-diffusion electrophoresis, the solute concentration gradient causes water to flow from the region of low solute concentration to the region of high concentration, inducing fluid flow and simultaneously causing the particles to move. The bubble recoil mechanism is caused by the accumulation of bubbles resulting from catalytic/non-catalytic reactions of particles in the fluid. Common microrobots that use the bubble recoil mechanism to achieve motion are mainly nanotubes. In addition, Janus particles can also be driven by the bubble recoil mechanism. In summary, chemically driven micromotors can be driven by different mechanisms. There are various chemical dyes used for chemically driven micromotors, mainly H_2_O_2_ [6,12,52], Br_2_ and I_2_ solutions [40], hydrazine [53], acidic and alkaline solutions [54,55], and so on. In addition, water can be used as a clean energy source to drive some micromotors. However, the limited lifetime of such micromotors limits their further development [50,56].

The actuation of microrobots by an applied energy field allows the directional navigation of microrobots in various types of fluids without the aid of chemical fuels. Since low-intensity magnetic fields cause little or no damage to biological tissues, and the motion of particles in the presence of magnetic fields is tunable [8,11], therefore, the use of magnetic fields to drive particles holds great promise for many biomedical applications. Magnetically driven miniature motors are expected to be used in the induction of neuron-like cell differentiation [5,9], cancer therapy [14,20], cell manipulation [15], drug delivery [3,4,30,57] and many other biomedical applications. In addition, magnetic micromotors can also be used in the field of environmental remediation [12,13]. As the size of the micromotors is further reduced or the distance between the particles and the applied magnetic field source is increased, the motion behavior of helically structured particles in vivo becomes more popular. Therefore, among the many structures of micromotors, magnetically driven micromotors are usually helical structures. Inspired by the flagellar drive of artificial bacteria, researchers have developed a large number of helical micromotors. They can convert rotational motion around the helical axis into translational rotational motion along the helical axis [9,30]. Ultrasound, as another biocompatible applied energy source, has been widely used in medicine. To date, researchers have developed a large number of ultrasound-driven micromotors [2,3,4,5,6,7]. On this basis, Park et al. [3] used ultrasound to drive a microrobot and demonstrated the effect of different drug release modes on the therapeutic effect of cancer cells. In addition, light and electricity can also be used as external energy sources to drive the particles, laying the foundation for the further development of multifunctional microbots.

The ultimate goal of the preparation is to create microrobots that can be used in various application scenarios such as biomedicine [2,3,4,5,17,20,21,23,24,27,52,58,59,60,61] and environmental remediation [19,62], among others. Therefore, methods to move particles should be developed for the characteristics of different types of microbots and the corresponding application scenarios. So far, particles have been able to perform various biomedical tasks by establishing appropriate stimulus–response mechanisms. For example, targeted drug delivery [63,64,65,66], neuron-like cell delivery and cell differentiation [5,9], biosensing [67], bioimaging [59], and early cancer diagnosis [33,34]. Meanwhile, recent findings have shown the great potential of particles in environmental monitoring and remediation processes [68,69].

**Figure 1:**
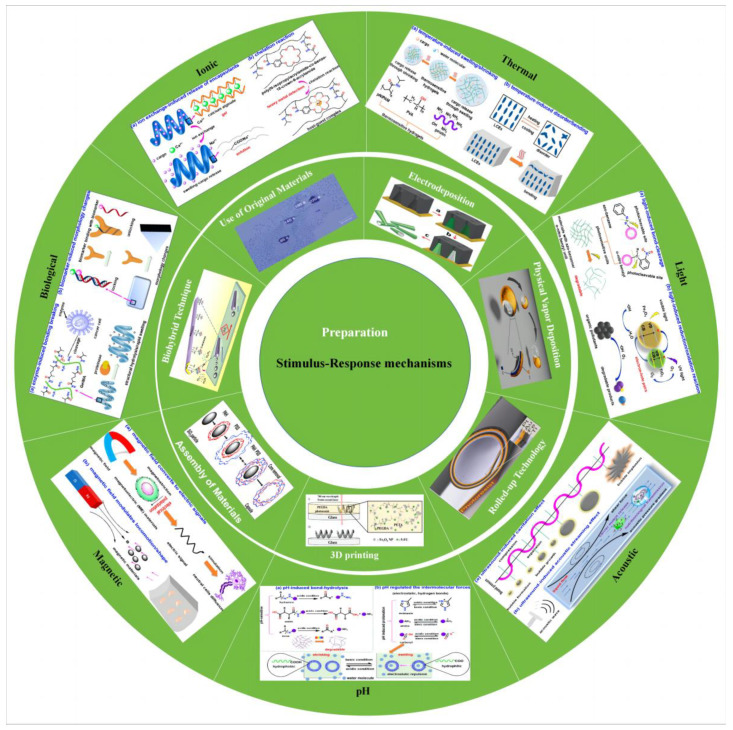
Preparation, stimulus–response mechanisms and applications of micro/nanorobots. Electrodeposition 77. Reproduced from Ref. [71]. Copyright 2011, the American Chemical Society; Physical vapor deposition 128. Reproduced from Ref. [72]; Copyright 2013, the American Chemical Society; Rolled-up Technology 97. Reproduced from Ref. [73]. Copyright 2009, Wiley-VCH; 3D printing technology 3. Reproduced from Ref. [3]. Copyright 2020, Wiley-VCH; Assembly of materials 153. Reproduced from Ref. [74]. Copyright 2012, the American Chemical Society; Biohybrid Technique 167. Reproduced from Ref. [75]. Copyright 2012, the American Chemical Society; Use of Original Materials 172. Reproduced from Ref. [76]. Copyright 2010, Wiley-VCH; Stimuli-responsive mechanisms 1. Reproduced from Ref. [1]. Copyright 2023, the American Chemical Society.

With the advancement of nanotechnology, methods for the preparation of micro- and nanorobots [70] and stimulus-responsive mechanisms [1] and their related applications have been successively reported. As shown in Figure 1, we summarize and demonstrate the various preparation methods for microbots and the stimulus response mechanisms. This review highlights the different methods of particle preparation and the factors that influence particle design, such as shape, composition and material distribution, followed by a description of stimulus-responsive mechanisms for microrobots and their applications. Finally, the current challenges facing microbots are highlighted and possible solutions are proposed to facilitate the practical application of microbots. By reviewing the research progress of artificial microrobots in recent years in terms of preparation methods, stimulus–response mechanisms and applications, we aim to illustrate the current opportunities and challenges facing artificial microrobots, and thus hope to provide inspiration for the development of novel preparation methods as well as actuation approaches.

## 2. Preparation of Microrobots

The emergence of different methods for creating adaptable micro- and nanorobots has been facilitated by the progress of nanotechnology. The key to making microengines is to break their original symmetry by design so that they can move under chemically or externally driven conditions to perform complex tasks. This section describes the preparation methods for producing micro- and nanorobots with different structures that can be used in different scenarios through different actuation methods (Table 1).

### 2.1. Electrochemical Depositions

Electrodeposition, also referred to as electrochemical deposition, enables the creation of microrobots with varying three-dimensional geometries using a range of metal and polymer materials. As such, electrodeposition is a viable method for producing microrobots. This technique does not necessitate costly equipment or rigorous experimental conditions and can be easily adapted to the needs of microrobots of various sizes. Typically, the material is deposited by applying an electric current, but it can also be executed through redox reactions.

#### 2.1.1. Membrane Template-Assisted Electrodeposition

Membrane template-assisted electrodeposition involves synthesizing various materials, including polymers, metals, semiconductors, and carbon, into desired tubular or nanowire structures using thin-film micropores [77,79,82,86,87]. These micropores act as reactors and facilitate the synthesis of required micromachines. With its monodispersed diameter and high micropore density, the membrane micropores are ideal for mass-producing microbots with similar nanostructures.

Typical membrane materials used are alumina membranes [116] and trace-etchable polycarbonate membranes [71]. The length of the prepared microrobots is proportional to the electrical charge and the diameter of the particles is matched to the diameter of the micropores. Depending on the chemical nature of the pore walls and the inherent properties of the material itself, the prepared nanostructures can be solid or hollow. Membrane-template-assisted electrodeposition provides an efficient and relatively low-cost method for the preparation of nanowires [83], nanotubes or helical micromotors. This section discusses the use of membrane-template-assisted electrodeposition for the preparation of nanowires, nanotubes and helically structured micromotors.

##### Electrodeposited Nanowires

**Figure 2:**
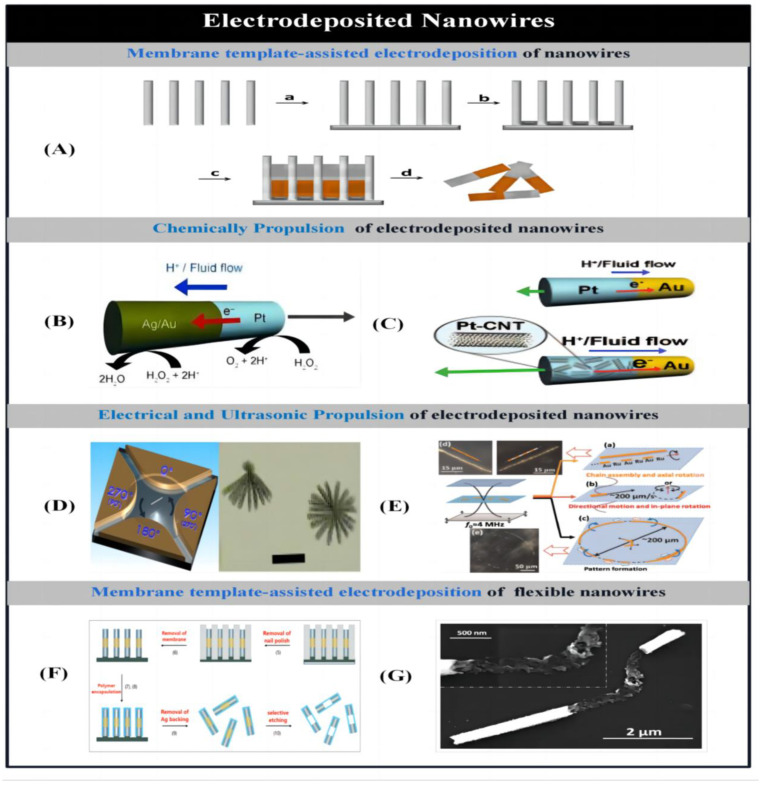
Modes of electrodeposited nanowires. (**A**) Membrane-template-assisted electrodeposition of nanowires: (a) deposition of gold or silver backing on the membrane template, (b) electrodeposition of sacrificial layer, (c) sequential electrodeposition of desired components, and (d) removal of the backing and sacrificial layer; dissolution of the membrane. Reproduced from Ref. [70]. Copyright 2015, the American Chemical Society. (**B**,**C**) Schematic representations of the self-electrophoresis mechanism of Ag-Au/Pt and Au/Pt-CNT nanowire motors in H_2_O_2_, respectively. Reproduced from Refs. [118,119]. Copyright 2008, Wiley-VCH and 2008, the American Chemical Society, respectively. (**D**) Rotation of nanowires by alternating current voltages applied to multiple electrodes: (a) schematic representation of experimental setup of quadruple electrodes and (b) images of free (right) and one end fixed (left) rotating Au nanowires. Reproduced from Ref. [87]. Copyright 2005, the American Physical Society. (**E**) Ultrasound-propelled nanowires: (a–c) illustration of the kinds of motion (chain assembly and axial spinning, axial directional motion and in-plane rotation, and pattern formation) of metal nanowires in an acoustic field; (d) and (e) dark field images of typical chain structures and ring patterns formed by Au and AuRu rods. Reproduced from Ref. [85]. Copyright 2012, the American Chemical Society. (**F**) Preparation procedure of flexible metallic nanowires with polyelectrolyte hinges after membrane template electrodeposition. Reproduced from Ref. [84]. Copyright 2007, Nature Publishing Group. (**G**) SEM image of a Au/Agflex/Ni nanomotor with flexible central silver segment. Reproduced from Ref. [82]. Copyright 2010, the American Chemical Society.

Paxton et al. [77] were the first to prepare bimetallic gold–platinum nanowires with a diameter of 370 nm and a length of 2 um using membrane-template-assisted electrodeposition, which would move along the axial platinum end direction in a 2–3% aqueous hydrogen peroxide solution (the velocity per second can be up to 10 times the body length). These bimetallic nanowire motors were mainly prepared by membrane-template-assisted electrodeposition. Subsequently, Fournier-Bidoz et al. [78] proposed an improved electrodeposition method for the preparation of bimetallic nanowire motors. A silver/gold layer was first deposited on one side of the membrane by physical vapor deposition to act as the working electrode. The membrane was then assembled in a Teflon plating bath and a flat aluminum sheet was placed on top of the metal layer to act as a conductive contact for subsequent electrodeposition. Typically, a copper or silver sacrificial layer is deposited first, followed by the deposition of the various desired metal materials. The silver/gold substrate and sacrificial layer are then removed by physical polishing or chemical etching, and the aluminum oxide membrane is placed in a solution of sodium hydroxide to dissolve it. After successive rinsing and centrifugation, the nanowire motor is released from the template and collected. It was used to prepare threaded rod-like nanowire motors by using an alumina membrane with nano-sized micropores into which different metals were sequentially deposited (Figure 2A). The nanorods were driven to achieve uniform circular motion by oxidative decomposition of hydrogen peroxide into oxygen at the unattached nickel end of the bimetallic nanorods. This bimetallic nanorod is self-powered by the catalytic decomposition of hydrogen peroxide into water and oxygen. The driving principle of the nanorods is based on a number of different mechanisms such as interfacial tension gradient, bubble recoil, viscous Brownian ratcheting and autoelectrophoresis to convert the chemical energy into mechanical energy required to move the system. Ambulo et al. [117] determined the potentials of the cathode and anode on each metal with equal reaction rates based on the Tafel plots obtained from the reaction of hydrogen peroxide on the cathode and anode on different metal ultramicroelectrodes. All possible directions of motion of the bimetallic assemblies were then predicted based on the electrochemical mechanisms of the bimetals, further confirming the bipolar electrochemical driving mechanism of the bimetallic nanorods.

Typically, the preparation process and nanowire structure are improved to produce these bimetallic nanowire motors with higher speed and efficiency. Demirok et al. [118] achieved a significant increase in the speed of fuel-driven nanowire motors (over 150 um/s) by replacing the cathodic pure gold segments with silver–gold alloy segments (Figure 2B). The catalytic activity and efficiency of the micromotor were further optimized by adjusting the alloy composition, including the spatial distribution of metals in the cathode segment. Based on the same principle, Laocharoensuk et al. [119] achieved a significant increase in the speed of the micromotor in a hydrogen peroxide solution by incorporating carbon nanotubes into the platinum component of the asymmetric metal nanowire motor (Figure 2C). Since the running speed of bimetallic nanomotors prepared by hydrogen peroxide fuel electrochemical deposition is related to the surface area of the catalytic segments of the nanorods, Zacharia et al. [120] increased the running speed of the nanomotors by increasing the surface area of the catalytic segments by introducing porosity and increasing the surface roughness of the catalytic segments. In addition to the use of commonly used hydrogen peroxide solutions to drive bimetallic motors prepared by electrodeposition, Liu et al. [40] introduced a highly efficient bubble-free nanomotor composed of copper–platinum segmented nanorods. The Cu-Pt nanorods acted as nano cells in dilute bromine or iodine solutions. The nanorods are moved with autoelectrophoretic action resulting from redox reactions generated at different metal segmentations. In aqueous bromine solution, the ionic gradient generated by the asymmetric dissolution of copper causes the asymmetric ratchet-shaped pure copper nanorods to undergo rotational and tumbling motions.

Membrane-template-assisted electrodeposition of nanowires can also be driven by applied acoustic, optical, magnetic, and electric fields, in addition to chemical fuels for self-propulsion. For instance, Fan et al. [87] induced the controlled and high-speed rotational motion of bimetallic nanowires by utilizing rotational forces generated by AC voltages applied on multiple electrodes (Figure 2D). Furthermore, Fan et al. [86] utilized an AC electric field applied to microelectrodes for actuating nanowires in a suspended state. These nanowires can be aligned in lines and chains, and can be propelled and assembled at a particular location either perpendicular to or parallel with their own orientation. Even at low Reynolds numbers, nanowires can diffuse under control. Suk et al. [121] described micro semiconductor diodes that were suspended in water and subjected to an AC electric field, which generated a localized electro-osmotic flow around the diode particles. Depending on the surface charge, the micro-semiconductor diode propelled the particles toward the cathode or anode.

Wang et al. [85] propelled metal nanowires, measuring 330 nm in diameter and 2 µm in length, through water or highly ionic solutions using ultrasonic standing waves at the MHz level. The acoustic field suspended, drove, rotated, aligned and assembled these metal nanowires (Figure 2E). These metal microrods are suspended at the cylindrical cell center plane upon tuning the ultrasonic frequency to a vertical wave. When utilizing continuous or pulsed acoustic waves, the metal microrods experience rapid axial motion at a rate of 200 µm/s at the resonant frequency. The directional movement of metal nanowires in the presence of an acoustic field is a result of the nanowires’ inherent asymmetry. This is caused by the nanowire ends being concave and convex, rather than flat during the electrodeposition preparation process. When ultrasonic waves are applied, the concave section collects the scattered energy of the sound waves, whereas the convex end reduces the energy density. This process produces acoustic pressure at the asymmetric ends, leading to propulsion. According to Nadal et al. [122], the propulsion of a microrobot under the influence of an acoustic field is attributed to the asymmetric steady fluid flow caused by the shape of the robot. To enhance the asymmetrical distribution of acoustic pressure and actuation properties of the particles, Garcia-Gradilla et al. [123] utilized spherical lithography to regulate the creation of concave cavities. In order to reinforce the concave cavities, polystyrene nanospheres were subsequently embedded into the nanopores of the silver-jet layer as a second sacrificial layer. After the deposition of various other metals, the nanospheres dissolved to form a concave cavity at the base of the nanowire.

Magnetic nickel nanowires prepared by membrane-template-assisted electrodeposition exhibit near-surface behavior [124]. Magnetic nanowires can not only move along arbitrary surfaces under the action of an applied rotating magnetic field, but also transport loads [83]. However, such magnetically driven rigid nanowires are limited by the working environment and mobility, making them difficult to apply to some desired scenarios. Therefore, Abbott et al. [125] proposed a novel magnetically driven flexible nanowire. It consists of a nickel head with magnetic properties and a flexible tail. When the applied magnetic field is aligned with the magnetic nickel head, the nanowire motor is decelerated and then propelled by the mechanical deformation of the flexible segment.

The preparation of flexible nanowires using membrane-template-assisted electrodeposition is similar to that of rigid nanowires. Only the additional step of using flexible polyelectrolyte multilayer segments (flexible hinges) to connect rigid nickel and platinum segments is added [84]. The flexible polymeric hinges were realized using polyelectrolytes to encapsulate the Ni/Au/Pt nanowires by electrostatic assembly of the layers. The flexible polymer hinge was then exposed with selective etching of the metal segments using potassium iodide solution (Figure 2F). In the presence of an applied fluctuating magnetic field, the nickel segments of the hinges are deformed, resulting in a radial forward fluctuating motion [126]. On this basis, Gao et al. [82] constructed flexible nanowires with bendable silver segments. Flexible gold–silver–nickel nanowires with fuel-free magnetic actuation were prepared by membrane-template-assisted electrodeposition. Partially dissolved and weakened silver bridges connect the gold head to the nickel tail. Under the action of an applied rotating magnetic field, the silver bridge generates cyclic mechanical deformation and also induces rotation of the nickel segments. The rotational action causes the gold segments to have different amplitudes so that the symmetry of the system is broken and motion is achieved (Figure 2G). The motion of the magnetically driven nanowires (forward or backward and start/stop of the motion) can be controlled by the length of the nickel and gold segments and by adjusting the magnetic field strength.

##### Electrodeposited Micro/Nanotubes

Segmented nanowires are driven in hydrogen peroxide solution by the bimetallic electrochemical degradation of a hydrogen peroxide solution produced by an autoelectrophoretic electrokinetic mechanism realized. The velocity of the nanowires and the resistivity of the solution satisfy a linear relationship. As the ion concentration increases, the speed of movement of the nanowires decreases, which can limit the application of chemically driven nanowires in environments with high ion concentrations [127]. To change this situation, Sanchez et al. [128] proposed a tubular micromotor. The inner surface of this micro-engine degrades hydrogen peroxide to produce bubbles that form a bubble recoil mechanism that drives the microtubes to move in an advective [111], helical [129] or circular [128], motion [130] at a higher speed. By adding a magnetic layer to the microtubules, multiple trajectories can be controlled and localized under a rotating magnetic field [73,131].

**Figure 3:**
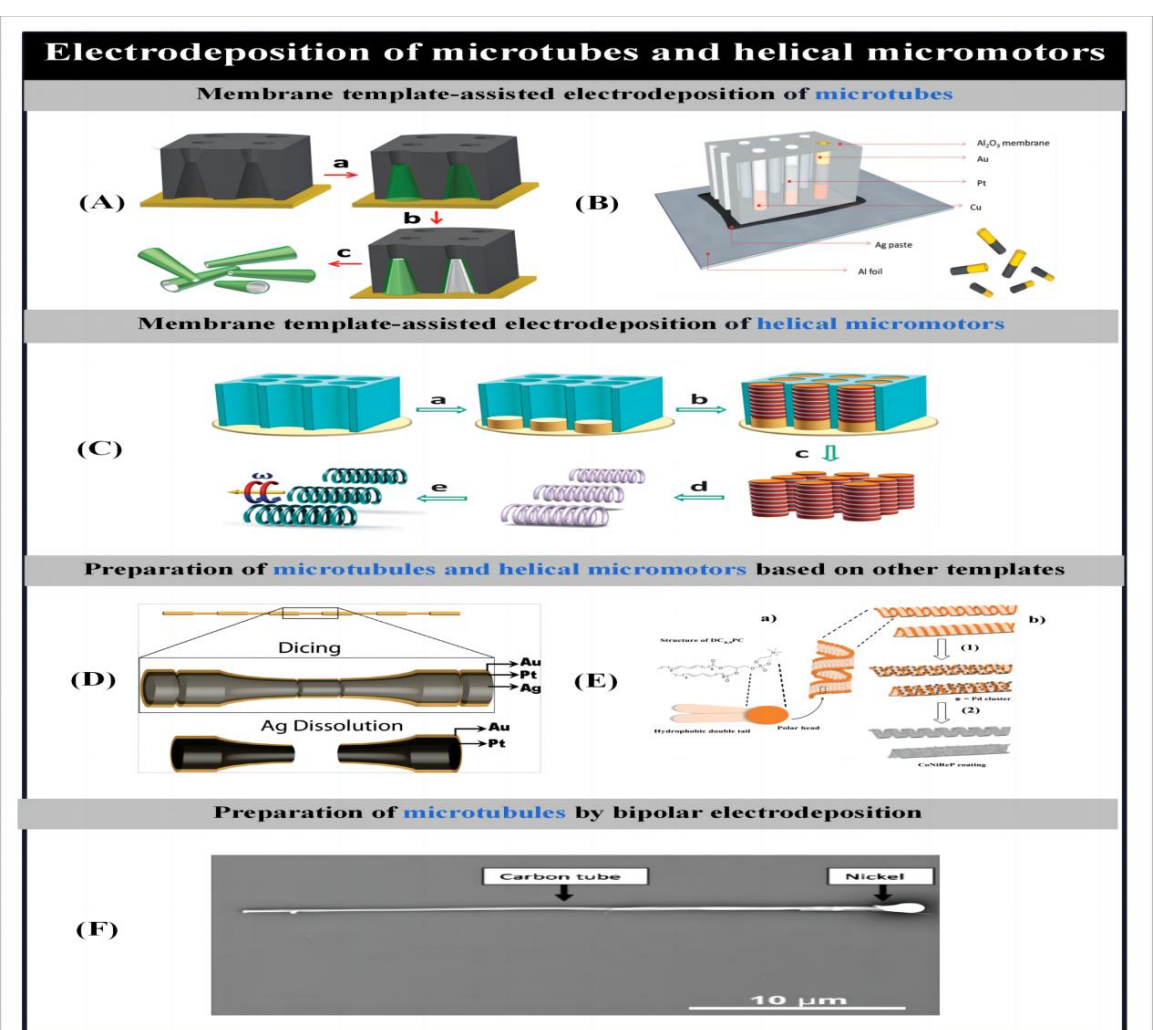
Modes of electrodeposited microtubes and helical micromotors. (**A**) Polycarbonate membrane-assisted preparation of conical PANI/Pt microtubes.(a) deposition of the polyaniline (PANI) microtube, (b) deposition of the Pt microtube, and (c) dissolution of the membrane and release of the bilayer microtubes.Reproduced from Ref. [71]. Copyright 2011, the American Chemical Society. (**B**) Anodized aluminum oxide (AAO) membrane-assisted preparation of segmented microtubes. Reproduced from Ref. [79]. Copyright 2013, the Royal Society of Chemistry. (**C**) AAO membrane-assisted preparation of helical micromotors. (a) Electrodeposition of Au; (b) electrochemical codeposition of Pd/Cu rods; (c) removal of the membrane template and the Au bottom layer; (d) etching of Cu; (e) Ni coating for magnetic actuation.Reproduced from Ref. [135]. Copyright 2014, the Royal Society of Chemistry.(**D**) Silver wire template-assisted layering approach for preparation of microtubes. Reproduced from Ref. [136]. Copyright 2010, the American Chemical Society. (**E**) Preparation of magnetic helical structure using lipids. (1) refers to the lipid surface activation by Pd cluster deposition, and step (2) represents the CoNiReP electroless coating..Reproduced from Ref. [80]. Copyright 2012, Wiley-VCH. (**F**) SEM image of nickel-modified carbon nanotube prepared by bipolar electrodeposition. Reproduced from Ref. [137]. Copyright 2010, Elsevier Ltd.

Similar to the preparation of nanowires using membrane-template-assisted electrodeposition, Gao et al. [71] prepared bilayer asymmetric polyaniline (PANI)/platinum microtubes using electrochemical growth of symmetric biconical microvias on a polycarbonate membrane template (Figure 3A). The plating bath assembly process was the same as that used to prepare the nanowires, where the aniline monomer was first electropolymerized due to its hydrophobicity and electrostatic effect. This was followed by the deposition of metallic platinum, resulting in the rapid synthesis of highly efficient catalytic microengines. The resulting microtubule engines are conical in shape, with the length and diameter depending on the size of the aperture of the thin film plate. These conical microtubules move ultra-fast (350 body length/s) in low concentrations of hydrogen peroxide solution.

In order to further optimize the composition and electropolymerizing conditions of polymer-based microtubular motors, Wei et al. [132] investigated the effects of material composition and electropolymerizing conditions on the actuation performance of polymer-based bilayer microtubular microrobots fabricated by a novel template. The effects of electropolymerizing conditions (monomer concentration and medium) on the morphology and kinematic properties of the microtubes were analyzed by comparing the effects of different polymerized outer layers and different metal inner surfaces on the motion of the bilayer microtubes. The most efficient propulsion was observed with PEDOT. The microtubules achieved a record-breaking speed of over 1400 body length/s in a physiological environment, the fastest speed of any man-made micromotor to date. An internal platinum–nickel alloy layer effectively combines magnetic guidance and catalytic fuel degradation in one layer, greatly simplifying the fabrication of microbots. The gold-coated polymer-based microrobot can be efficiently biocatalytically powered in a low concentration hydrogen peroxide solution in conjunction with catalase.

To replace the platinum-catalyzed decomposition of hydrogen peroxide as an energy source for microrobots, Gao et al. [54] described the effective autonomous motion of tubular (polyaniline) PANI-Zn-based microrockets in strongly acidic environments without the need for additional chemical fuels. The acid-driven, hydrogen bubble-propelled microrockets were fabricated using conical polycarbonate templates. Self-propulsion in acidic media is achieved by propulsion by hydrogen bubbles generated by simultaneous redox reactions on the internal zinc surface. And based on the driving characteristics of the microrockets in different acidic media and in human plasma, the velocity–pH dependence of pH measurements under strong acidic conditions was obtained. In addition to polymer-based tubular microengines, complete concentric metal microtubes can also be fabricated using the membrane-template-assisted electrodeposition method [133]. It simplifies the fabrication process by using graphite colloids instead of deposited metal substrates, thus avoiding the jetting process of physical vapor deposition. In contrast, Zhao et al. [79] fabricated threaded metal nanotubes with a diameter of 300 nm by electrodeposition of silver ink on an anodic alumina membrane template using aluminum foil as the working electrode. In contrast to concentric wound microtubes, this nanomicrotubule has a peculiar segmented structure in which different metals are arranged longitudinally (Figure 3B).

##### Electrodeposited Helical Micromotors

Liu et al. [134] prepared palladium nanosprings by anodic alumina membrane-assisted electrochemical deposition using nanochannels. The hydroxyl-terminated surface of the nanochannels can selectively absorb hydrogen ions to form a compact layer in the presence of a suitable pH, the presence of an effective potential, and an electroplating solution consisting of PdCl_2_, CuCl_2_ and HCl. The terminal hydroxyl surfaces of the alumina nano-channels and localized hydrogen precipitation contribute to the growth of palladium atoms at the outer sites of the alumina nano-channels. Palladium is automatically wound onto copper nanorods by helical dislocation, and palladium nanosprings are obtained after the selective removal of copper. Using this approach, Li et al. [135] prepared very small but highly efficient helical magnetic nanowires by template electrosynthesis. The helical magnetic nanowires can be efficiently driven under a low rotational magnetic field (Figure 3C). Palladium–copper nanorods were fabricated into nanoporous thin films by template electrosynthesis, after which the copper was removed. A magnetic nickel layer was deposited on the palladium nanospiral structure using an electron beam, and then the palladium nanospiral structure was obtained. When the helical nanomotors were prepared by the template-assisted electrodeposition method, the diameter and length of the micromotors could be adjusted by adjusting the diameter of the nanopores and the ion concentration in the plating solution.

#### 2.1.2. Electrochemical Deposition Based on Other Templates

The templates used for electrochemical deposition can not only be porous membranes, but also the shape of the template can be chosen according to the structure to be prepared. On the other hand, Manesh et al. [136] used a simplified template-supported layer to prepare catalytic conical tubular microengines. The templates were diced and dissolved by sequential deposition of platinum and gold on etched silver lines (Figure 3D). This approach allows control of the tubular engine parameters and improves the performance of the micro-engine. The micro-engine is actuated by the formation of a bubble recoil mechanism by internally generated oxygen microbubbles, producing a salt-independent motion that breaks the ionic strength limitations of the catalytic nanowires. However, this method is not suitable for batch preparation and the speed of the nanotubes produced is relatively limited.

Schuerle et al. [80] used self-assembled tubes and helical structures of DC_8,9_PC as templates. Magnetic manipulation was achieved by converting the lipid scaffold structures into rigid helical or hollow tube shapes that could be magnetically manipulated after coating with a uniform alloy. In this case, the microstructures of tubular liposomes were obtained by diffusion of DC_8,9_PC in an aqueous solution of ethanol after heating and cooling to chain melting temperature. Helical liposomes were formed by adding water in ethanol. The resulting liposomes were activated by chemically deposited platinum clusters to maintain the shape, and then the resulting rigid structures were placed in a CoNiReP plating solution to coat them with an alloy layer for magnetic manipulation (Figure 3E).

#### 2.1.3. Asymmetric Bipolar Electrodeposition

Janus particles, as a special type of nanoparticle with asymmetric surfaces, have significantly different physical or chemical properties and orientations. In general, asymmetric chemical electroplating is used to prepare Janus particles [89]. Bipolar electrochemistry refers to the fact that when a conducting substance is placed in a strong electric field between two electrodes, a potential difference is created between the two ends of the substance. The polarization intensity of the conducting substance is proportional to the strength of the electric field and the characteristic dimensions of the substance, and it produces asymmetric activity on the surface of the substance in a wireless manner. When the polarization strength is high enough, an electrochemical reaction occurs at both levels of the substance, breaking the symmetry of the substance and producing two-sided properties.

A variety of materials including metals, semiconductors, insulators and molecular layers have been used to produce Janus particles of different sizes and shapes. The bipolar electrodeposition of metals generally involves applying a perpendicular electric field to the particles. By inducing a redox reaction at the poles of the particles, metal salts are reduced at the cathode and metal deposits are formed on the surface of the particles. Catalytic and magnetic Janus particles prepared by bipolar electrochemistry can be used as self-propelled or magnetically driven micromotors. For example, Loget et al. [137] used the technique of bipolar electrochemical deposition to prepare asymmetric nanoparticle–carbon microtubules. A suspension containing carbon microtubules was added to a capillary containing a nickel salt solution, and then a high intensity electric field was applied to orient and polarize individual microtubules. The ends of the microtubules are the sites of water oxidation and nickel ion reduction, and the water is oxidized to produce gas bubbles to form a bubble recoil mechanism. If the polarization strength of the microtubules is high enough, they are driven through the capillaries by the bubbles, and the nickel deposited at one end of the microtubules causes the microtubules to achieve directional motion under magnetic field manipulation (Figure 3F). This method of bimetallic electrochemical deposition using a cell avoids the fixation of particles on the surface. In addition, the deposition structure can be adjusted by changing the direction and amplitude of the electric field and the viscosity of the medium.

The electrochemical method offers a unique opportunity for preparing nanomaterials. This approach is characterized by simple equipment, convenient operation, and low energy consumption. The dimensions of these materials can be customized to specific needs. Additionally, it allows for the obtaining of nanomaterials in various shapes and sizes by altering the pore size of the template and electrochemical parameters. The method has a broad range of applications and can be used with any species that can be deposited on an electrode to prepare nanoparticles. It can also be combined with other techniques. However, the electrochemical synthesis of nanomaterials is limited to objective evaluations and requires clear, concise language without biased or emotional terminology. However, the electrochemical synthesis of nanomaterials began late, and the mechanism of some reaction processes remains unclear. Additionally, current methods cannot produce nanomaterials in large quantities. Therefore, further research is necessary.

### 2.2. Physical Vapor Deposition

Physical vapor deposition is used to deposit thin layers of material as a vapor coating. The solid material is evaporated using a high temperature vacuum or gaseous plasma and the vapor is then transported to the surface of the vacuum substrate where it condenses to form a thin film. There are typically two types of physical vapor deposition: sputtering and electron beam evaporation. Sputtering involves the use of plasma gas to bombard a solid target material to vaporize it, whereas electron beam evaporation uses an electron beam to vaporize atoms from a target state into a gaseous state. The gaseous state produced by either technique is then deposited onto the metal surface. This section describes conventional physical vapor deposition and modified swept angle deposition.

#### 2.2.1. Conventional Physical Vapor Deposition

**Figure 4:**
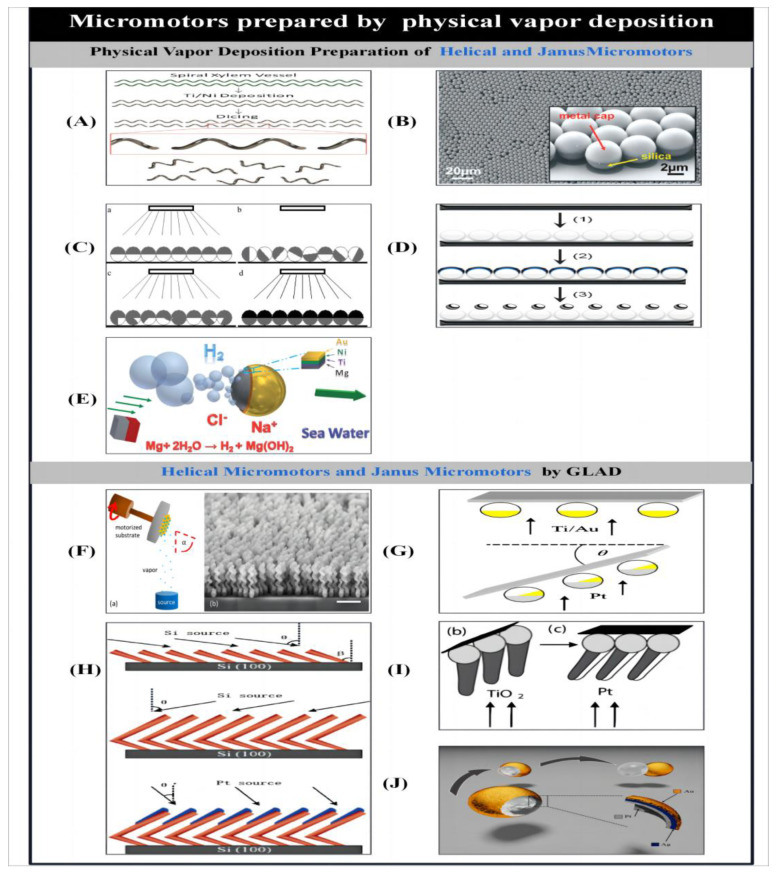
Micro/nanomotors prepared by conventional physical vapor deposition. (**A**) Plant-based helical micromotors prepared through physical vapor deposition. Reproduced from Ref. [81]. Copyright 2013, the American Chemical Society. (**B**) SEM image illustrating an array of self-assembled spherical particles. The inset shows the coating of the Janus particles with metal film providing its catalytic and magnetic properties. Reproduced from Ref. [46]. Copyright 2012, the American Chemical Society. (**C**) Schematic of fabrication of bimetallic Janus micromotors by conventional physical vapor deposition. (a) halfcoated with one metal. The spheres are then resuspended in water and (b) deposited again with random orientation. (c) The exposed uppersurfaces are coated again. This process is repeated until the sphere is completely coated with the first metal, and then the spheres are halfcoated with a second metal. Reproduced from Ref. [142]. Copyright 2010, the American Chemical Society. (**D**) Formation of sphere dimers via thermal annealing. (1) Silica sub-monolayer deposition over the substrate. (2) Metallic half-shell deposition over the silica beads: a 5-nm chromium adhesion layer was first sputtered followed by a platinum thicker layer. (3) Thermal annealing at 900 8C for 3 h. Reproduced from Ref. [143]. Copyright 2009, Wiley-VCH. (**E**) Schematic diagram of Mg-based seawater-driven Janus micromotor. Reproduced from Ref. [56]. Copyright 2013, the Royal Society of Chemistry. (**F**) Schematic diagram of GLAD technique and SEM image of the helices prepared by this method. (a) Schematic of the GLAD technique and (b) SEM images of a wafer containing helices representative of those used in this work. Reproduced from Ref. [150]. Copyright 2013, the American Chemical Society. (**G**) Preparation of asymmetric Pt/Au-coated catalytic micromotors by GLAD. Reproduced from Ref. [151]. Copyright 2010, the American Institute of Physics. (**H**) Fabrication procedure of L-shaped Si/Pt nanorod motors by GLAD. Reproduced from Ref. [152]. Copyright 2007, the American Chemical Society. (**I**) Synthesis of catalytic micromotor consisting of a spherical silica colloid with a TiO_2_ arm coated asymmetrically with Pt. Reproduced from Ref. [153]. Copyright 2009, Wiley-VCH. (**J**) Pt–Ag–Au shell micromotor fabricated by GLAD. Reproduced from Ref. [72]. Copyright 2013, the American Chemical Society.

Physical vapor deposition directly transforms the target material into microstructures with the desired properties. Inert materials can be introduced into the process to catalyze or block reactions, and magnetic materials can be introduced to allow directional movement in the presence of a magnetic field. In the manufacture of magnetically actuated micromotors, a template suitable for magnetic actuation is first prepared and then the magnetic material is deposited using physical vapor deposition. Templates for magnetic actuation can be prepared with various methods such as swept angle deposition, 3D laser direct writing or directly using naturally occurring structures. Gao et al. [81] introduced a magnetically actuated helical microswimmer. This helical microstructure is a helical water-conducting vessel from various plants, and the geometric variables of the helical vessel (helix diameter and pitch) can be controlled using mechanical stretching. Thin layers of titanium and nickel are deposited directly onto the spiral vessels by electron beam evaporation for dicing, enabling the mass production of versatile spiral microswimmers (Figure 4A). In addition to using magnetic forces and moments to actuate magnetic materials, Baraban et al. [91] used magnetic field-induced thermophoresis to enable the movement of silicon spheres with magnetic caps. Using magnetic spherical Janus micromotors fabricated by physical vapor deposition, the particles were induced to thermophoretically move by heating their magnetic caps with an AC magnetic field. At the same time, a DC magnetic field was used to orient the Janus micromotors and guide them into long time scale motions. The Janus micromotors are highly bioadaptable because they no longer use chemical fuels. The special properties of the ultra-thin 100 nm thick polymerized magnetic film allow the magnetic cap structure of the Janus micromotor to have a topologically stable magnetic vortex state, allowing full control of the motion.

To enable nanowires prepared by template-assisted electrodeposition to move from simple linear motion to free rotational motion. Qin et al. [138] fabricated gold–platinum–gold tri-segmented nanorotors (360 nm diameter; 1.67 μm length of gold in the first segment, 3.33 μm length of platinum, and 20 nm passivation cross section length of gold in the end segment) by photolithography within nanowires using template-assisted electrodeposition. The symmetry of the nanorotors was broken by depositing a gold–chromium bilayer coating on the end-gold surface using thermal deposition. Propulsion of the nanorotors was achieved via the dynamic decomposition of hydrogen peroxide in hydrogen peroxide solution to generate bubbles. Subsequently, Wang et al. [139] prepared bimetallic Au-Ru nanorods using an anodic aluminum oxide film. After releasing the bimetals from the alumina film, chromium, silica, chromium, gold and platinum were sequentially deposited on each side of the rods. By breaking the original symmetry of the nanorods, they can be driven by decomposing hydrogen peroxide. In addition, the additional layers of gold and chromium provide vertical forces that move the rod towards the center of the orbit due to the asymmetric flow.

In addition, bipolar electrochemical deposition is used to create asymmetry. If the orientation of the substrate is not changed, the material introduced by physical vapor deposition is only deposited at one end of the material. In this way, Janus micromotors with asymmetric structures can be produced. The key to producing Janus micromotors using physical vapor deposition is the asymmetric distribution of the catalyst or reactant. This means that the environment in which it is placed should contain a suitable substrate (hydrogen peroxide). The non-uniform consumption of the substrate and the non-uniform distribution of the reactants allow the micromatter to degrade the hydrogen peroxide by decomposition. The chemical concentration gradient created during the degradation process allows the flow of fluid around the microparticles to achieve actuation of the particles [140]. In the preparation of Janus microengines, each side of the particles should be selectively synthesized to give them different properties. In the preparation of Janus micromotors using physical vapor deposition, the symmetry is usually broken with a temporary shield of a hemisphere.

In the preparation of catalytic Janus micromotors, physical vapor deposition is usually used to deposit catalytic materials on the hemispherical surfaces of the particles, giving the Janus micromotor an asymmetric nature. For example, Baraban et al. [46] formed self-assembled monolayers of particles by the bottom-up deposition of suspended droplets of particles in a matrix followed by slow evaporation (Figure 4B). In order to slow down the evaporation process, smaller, inclined boxes are typically used for the evaporation process to ensure that an ordered array of particle monolayers is obtained [141]. The area of the resulting monolayer can be controlled by varying the particle concentration and droplet size of the suspension. The catalytic material is deposited onto the monolayer, forming a half-shell on the surface of the particles, which is then separated from the substrate by ultrasound.

The most common catalytic nanomotors are platinum-catalyzed Janus particles. The non-uniform consumption of catalysts and the inhomogeneous distribution of reactants are used to allow the microscopic substances to degrade hydrogen peroxide. The degradation process creates a chemical concentration gradient that causes the fluid to flow around the particles, thus enabling the particles to be driven [140].Wei et al. [53] proposed another type of Janus particle with spherical diffusive motion. The iridium-based catalytic Janus micromotor was prepared by physical vapor deposition of iridium onto silica particle hemispheres. The Janus micromotor relies on the iridium hemispherical layer to catalytically degrade hydrazine attached to the silica spherical particles, and the resulting permeation effect allows the micromotor to be self-driven at very high speeds with very low concentrations of hydrazine fuel. The concentration of chemical fuel required to drive the micromotor in this way is reduced by a factor of nearly 10,000 compared to driving a conventional catalytic micromotor. Based on the same principle, Hong et al. [92] advanced the particles by irradiating silver-coated colloidal beads with UV light, resulting in the asymmetric release of Ag^+^ and OOH^−^. This manipulation demonstrated that the macroscopic order we observe is derived from the disordered behavior of microscopic matter. Subsequently, Wheat et al. [142] used physical vapor deposition to fabricate bimetallic spherical micromotors. After spraying a layer of metal onto the surface of the microspheres, another metal was sprayed onto the half surface of the coated metal. This spherical bimetallic motor induced a charge autoelectrophoresis mechanism by reaction. Self-actuation was achieved by the electrocatalytic degradation of hydrogen peroxide, and the speed of movement was consistent with that of rod motors of the same size and composition (Figure 4C).

Based on spherical templates, physical vapor deposition can also be used to fabricate micromotors of various structures. Valadares et al. [143] fabricated self-powered spherical dimers using physical vapor deposition. Submonolayer silicon microspheres, a thin adhesive chromium layer and a thicker adhesive platinum layer were first sequentially deposited in a silicon/platinum matrix. The metal half-shells were then dehumidified by an annealing process to attach non-catalytic silicon spheres to catalytic platinum spheres (Figure 4D). In addition, to make the micromotor move without the use of an applied field, it is necessary to add asymmetry to the particles, either in their chemical composition or in their physical geometry. Zhao et al. [144] fabricated coconut-like micromotors by partially or completely etching silica templates from platinum. Although the inner and outer surfaces of the micromotor were made of the same material, structural motion was also possible because bubbles could be generated on the convex surface. It was shown that the micromotors could be driven not only by chemical asymmetries but also by geometrical asymmetries, and that the partially etched motors were faster than the fully etched or Janus micromotors. Tierno et al. [145] prepared extended ellipsoidal microspheres with catalytic properties by coating the half surfaces of the particles with platinum particles by vacuum sputtering.

The alternative drive mechanism for Janus micromotors is non-catalytic. Because the materials that make up the motor are consumed during the actuation process, this non-catalytic micromotor has a size of a few tens of microns. The actuation is achieved by forming an inert layer on the surface through physical gasification deposition, which creates asymmetric bubbles. To make the non-catalytic micromotor operable with nearby fuels, micromotors are generally fabricated using materials that can react with water. For example, Gao et al. [50] used physical vapor deposition to fabricate Janus micromotors. Instead of using normal hydrogen peroxide as a fuel, it is self-powered by a bubble recoil mechanism generated by water actuation. The micromotor consists of microspheres partially coated with an aluminum–gallium dual alloy, consisting of a mixture of aluminum particles and liquid gallium microcontacts. Bubbles are generated at one end of the exposed aluminum–gallium alloy hemisphere, which collide with the water to provide forward propulsion. Meanwhile, Gao et al. [55] presented micromotors that achieve propulsion by capturing energy from the reaction of three different chemical fuels (acid, alkali, and H_2_O_2_). This aluminum–palladium Janus micromotor is achieved by the deposition of palladium on one side of aluminum particles. Propulsion is achieved by hydrogen bubbles generated by various chemical reactions of aluminum in strong acid and alkaline environments; the oxygen bubbles are generated by the palladium coating in the hydrogen peroxide medium. This method of preparation achieves high speed and long duration of propulsion of the particles in acidic and alkaline media.

Another material used to make non-catalytic micromotors that can react with water is magnesium. Gao et al. [56] described magnesium-based micromotors powered by chloride-containing seawater (Figure 4E). A nickel–gold bilayer patch was deposited onto magnesium particles using electron beam evaporation, creating an inert metal cap that could generate asymmetric thrust. The reaction of magnesium with water was promoted using high current corrosion and chloride ion pitting processes, enabling the micromotor to be self-powered. Similarly, Fangzhi et al. [146] presented a novel biocompatible magnesium–platinum micromotor. It was powered by a magnesium–water reaction using sodium bicarbonate. Sodium bicarbonate forms magnesium carbonate in molten water by reacting with magnesium hydroxide, a passivated layer on the surface of magnesium, which facilitates the generation of asymmetric magnesium–water reaction bubbles and thus propels the microbot.

#### 2.2.2. Glancing Angle Deposition

Conventional physical vapor deposition substrates are typically placed parallel to the target and the gas stream is then deposited from the target perpendicular to the substrate. Grazing angle deposition (GLAD, also known as dynamic shadowing growth), on the other hand, deposits the gas stream onto the substrate in an oblique manner. To achieve geometric shadowing, the substrate is typically placed at an inclined angle (relative to the airflow volume). By rotating the substrate in polar and azimuthal directions, different desired nanostructures can be produced, for example, nanorod arrays with different shapes, nanospring arrays or even multi-layered nanostructures [147]. Microrobots with complex 3D structures can be fabricated in bulk using swept angle deposition, and this section focuses on the fabrication of spiral and Janus micromotors using swept angle deposition.

##### Helical Micro/Nanomotors by GLAD

Helical growth can be achieved by rotating the azimuthal angle of the tilted substrate during deposition. In order to produce helical structures with good homogeneous properties on a large scale and in bulk, the substrate must be crystal seeded prior to the GLAD process. Since the size, shape and uniformity of the crystalline seed have a large influence on the properties of the resulting helical structures, an ordered array of crystalline seeds is generally prepared on the surface of the substrate. Ghosh et al. [94] described the construction and manipulation of helical chiral gel drivers. The desired helical structure was formed by depositing a stream of silica at a given angle of inclination onto silica microbeads in a rotating matrix. Cobalt is then deposited on the outer surface of the helical structure by thermal evaporation. The helical microstructures can be stimulated by magnetic fields of the same type to achieve micrometer precision navigation in water. The size of the helical structure depends on the size of the crystalline species. In general, gold nanodots prepared by micellar nanolithography are usually used as seeds to reduce the size of the helical structures [95]. Self-assembled AuCl_4_ bi-block copolymer micelles were spin-coated onto the wafers to form a homogeneous mono-micellar film. The subsequent plasma treatment to remove the polymer while reducing the gold salt content resulted in regular arrays of gold nanodots. The spacing and size of the nanodots were controlled by varying the molecular weights of the diblock copolymers and gold loadings, and the spin-coating speed. This helically structured nanodriver is embedded with filaments 70 nm in diameter. As the size of the nanodriver is smaller than previous nanodrivers and detachable microorganisms, therefore, Brownian forces in pure water can hinder its movement, but it can still pass through highly viscous solutions at speeds comparable to those of large microswimmers.

Ghosh et al. [148] investigated the minimum size limits of magnetically driven helical motors using experimental observations and numerical modelling. The results show that on small-length scales, directional noise can have a significant effect on the direction and size of the helical drive motion. At length scales smaller than a few microns in aqueous media, the drive system must operate at a frequency that is the inverse cube of its size due to the limitations of the helical drive in achieving directional motion. At the nanoscale, motion in the fluid is mainly controlled by viscous drag. An effective propulsion method for fuel-free actuation in complex media is the use of weak rotating magnetic fields to drive helical microrobots. Based on the same principle, Walker et al. [149] demonstrated the optimal length of the microdrive (very short, only one helical loop) by combining analytical and numerical theory with experiments on nanostructured ferromagnetic helical drives.

The grazing angle deposition technique allows not only the growth of complex three-dimensional structures containing different metals such as metals, insulators, semiconductors and magnetic materials, but also the production of binary alloys by simultaneous deposition in two evaporators. The alloy composition can be adjusted by individually controlling the deposition rate of each evaporator. During the growth process, ferromagnetic nickel segments can be added to the helical structure. Instead, a thin magnetic layer is deposited on the helical structure released from the circular wafer, allowing reproducible production of magnetically driven helical motors with good uniform magnetism. The chirality and pitch of the helical structure can be controlled by changing the direction and speed of rotation of the substrate during the deposition process. However, the physical properties of chiral molecules are weakened by the destruction of the asymmetry. To study the physical properties of chiral molecules, complex non-spherical colloidal particles can generally be used as colloidal molecules in macroscopic model systems to visualize molecular phenomena that are difficult to observe. However, since surface minimization favors the growth of symmetric particles, making it difficult to synthesize chiral colloidal molecules, Schamel et al. [150] used swept angle physical deposition to mass produce chiral molecules and investigated the propeller effect. By coupling the dipole moments of the spinning enantiomers through an applied rotational field, the exocyclic mixture could be separated while being helically driven in the opposite direction (Figure 4F).

##### Janus Micro/Nanomotors by GLAD

Based on the self-growth effect and substrate rotation of GLAD, it can be used to easily fabricate Janus micromotors with complex structures. Gibbs et al. [151] used GLAD to fabricate asymmetric Pt-Au-catalyzed micromotors. Asymmetric bimetallic coatings were obtained after deposition of bonded titanium and gold. The substrate with silicon microspheres was rotated to a polar angle, followed by platinum deposition to expose the gold (Figure 4G). The micromotor was driven by an autoelectrophoretic mechanism generated by the degradation of hydrogen peroxide fuel in solution, and the average velocity u over the exposed gold surface area A was u ∝ A3/2. This catalytic micromotor has a higher activity than the micromotor driven by a non-autoelectrophoretic mechanism of the same size and morphology. Its motor behavior can be tuned by changing the overlap of the two Pt-Au metals. Similarly, Lee et al. [38] fabricated Pt-Au Janus micromotors by deposition of gold swept onto arrays of Pt nanoparticles prepared by micellar lithography of block copolymers under rapid substrate rotation. The size of the particles was comparable to some enzymes (30 nm), and this micromotor also catalytically degraded hydrogen peroxide to water and oxygen, which was self-driven by an autoelectrophoretic action.

Yuping et al. [152] prepared catalytic nanomotors with different geometries and controllable motions using GLAD based on the shadow effect and substrate rotation. Examples include rotating silicon–platinum nanorods, rotating L-shaped silicon–platinum or silicon–silver nanorods, and rotating silicon–silver nanosprings. A silicon nanorod skeleton is first prepared using GLAD, and then platinum or silver is asymmetrically deposited on one end of the nanorod skeleton by geometric shading. L-shaped nanorods were obtained by subjecting the substrate to rapid azimuthal rotation at the center of the tilt angle deposition (Figure 4H). Complex rotatable silicon–silver springs were fabricated by further controlling the deposition angle and substrate rotation speed. Gibbs et al. [153] fabricated multi-component rotatable nanomotors by growing platinum-coated titanium dioxide nano-arms on micron-sized silica microbeads via GLAD. The arms of the micromotor were deposited on the densely packed microbeads at an inclined angle and then platinum was deposited at an angle on only one side of the arm to create asymmetry (Figure 4I). Thus, when placed in a hydrogen peroxide solution, the structure rotates around an axis through the center of the beads and perpendicular to the nano-arms. By tilting the direction of the air flow and rotating the substrate, the resulting deposition layer can cover most of the area of the spherical template. Huang et al. [72] fabricated a Pt-Ag-Au shell micromotor (Figure 4J) by combining physical vapor deposition and wet chemical etching. The catalytic coating inside the shell can decompose hydrogen peroxide to produce gas bubbles. However, due to the low nuclear energy of the bubbles themselves, they do not block the openings in the shell and the micromotor achieves actuation under the bubble ejection or bursting mechanism.

Physical vapor phase deposition is a frequently used technological tool in the preparation of microrobots. This is accomplished through a physical vapor phase deposition process wherein controlled deposition of atoms or molecules in the gas phase leads to the formation of thin films or nanostructures on the substrate’s surface. Physical vapor phase deposition offers crucial advantages. Firstly, physical vapor phase deposition offers a high degree of control, allowing for precise regulation of the morphology, size and composition of micro- and nanostructures through the adjustment of parameters such as deposition conditions, gas phase flow and deposition time. Secondly, physical vapor phase deposition facilitates the preparation of high purity materials, leading to reduced impurities and the enhanced performance and stability of micro- and nanorobots. Moreover, it offers the adaptability of various material options to fabricate a diverse array of materials, encompassing metals, semiconductors, oxides, among others, with promising potential for varying applications. Additionally, physical vapor phase deposition is conducive to the extensive and sequential growth of slender coatings or nanostructures, which is suitable for the ample-scale production of micro- and nanorobots. However, the production of micro- and nanorobots via physical vapor phase deposition also presents several drawbacks and limitations regarding current technology. Firstly, more advanced equipment and vacuum systems are necessary, elevating the complexity and cost of the preparation. Secondly, substrate materials for physical vapor deposition require a relatively unique thermal stability and compatibility, limiting the selection of the preparation. Moreover, the high deposition temperatures required in the preparation process can restrict the usage of temperature-sensitive materials and specific applications. Furthermore, the crystal structure imposes limitations on the growth direction of physical vapor deposition, leading to challenges in achieving complex structural and morphological control.

### 2.3. Rolled-Up Technology

The convolution process uses stress design to transform nanofilms into three-dimensional structures such as wrinkles, tubes and spirals. By adding a stress gradient to the deposited film, it allows the film to form the desired nanostructure when released from the substrate. Currently, the convolution process is mainly used to produce tubular and helical micromotors.

#### 2.3.1. Preparation of Nanotubes Using Rolled-Up Technology

**Figure 5:**
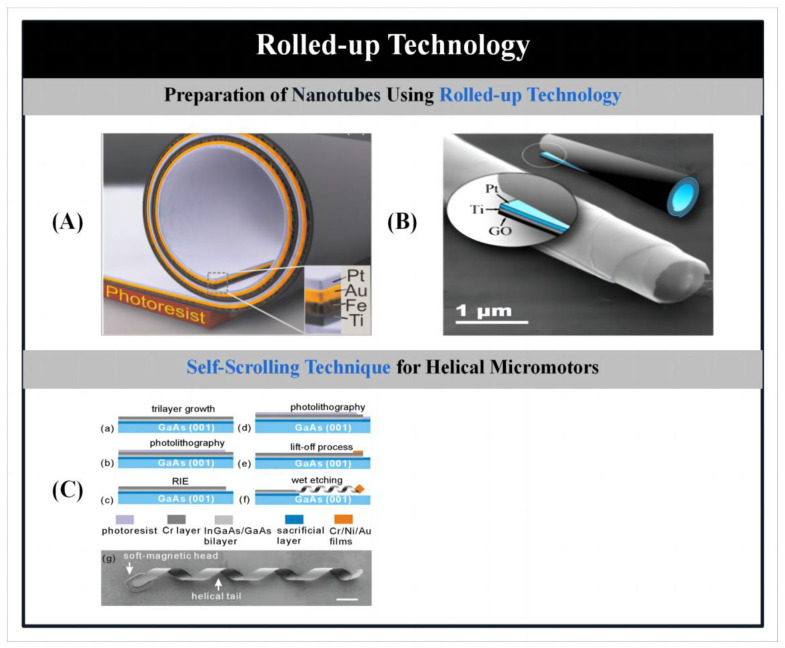
Micro/nanomotors prepared by rolled-up technology. (**A**) Schematic diagram of a rolled-up microtube consisting of Pt/Au/Fe/Ti multilayers on a photoresist sacrificial layer and an array of rolled-up microtubes. Reproduced from Ref. [73]. Copyright 2009, Wiley-VCH. (**B**) Rolled-up microtubes with graphene oxide as an external layer. Reproduced from Ref. [156]. Copyright 2012, the American Chemical Society. (**C**) Fabrication procedure of helical micromotors using self-scrolling technique. (a–f) Fabrication procedure of the ABF with InGaAs/GaAs/Cr helical tail. (g) SEM image of an untethered ABF. The scale bar is 4 μm. Reproduced from Ref. [97]. Copyright 2009, the American Institute of Physics.

Solovev [73] and Yongfeng et al. [154] were the first to use the convolution process to produce nanotubes. The microtubes consisted of an inner platinum layer (catalytic layer), an outer iron layer (magnetic remote control), and titanium and copper layers to connect the inner and outer layers and facilitate controlled rolling. The prestressed nanofilms were first deposited onto a photoresist sacrificial layer using photolithography, followed by the selective etching of the sacrificial layer using acetone. Tilt deposition using physical vapor deposition was used for precise positioning to integrate the tubes on a single wafer. The stress gradient required for the convolution process is then created by carefully controlling the substrate temperature, rotation rate and stress changes during deposition. Finally, the photoresist is etched away to release the pre-stressed polymetallic film deposited on the sacrificial photoresist layer from the substrate surface, and the film is automatically convolved to form a tube structure (Figure 5A) [73]. To avoid shape collapse of the convolved nanofilms, the microtubes must be dried using a critical point drying technique. The outer layer of these microtubules has a diameter range of 1–30 μm, which can be tuned by varying the thickness of the nanofilm and the built-in stress [155]. In general, the length of the microtubules produced by convolution is in the range of tens of microns. During the preparation process, a catalyst such as platinum is simply deposited on the top of the nanomembrane, which is folded to form the inner wall of the microtubule. During the release process, the folding orientation of the release film is determined by the crystal structure of the sacrificial layer and different etching rates along the crystal axis.

To simplify the complex convolution process, Kun et al. [156] prepared microtubes with an outer layer of graphene oxide by depositing a bimetallic layer on a single graphene oxide nanosheet (Figure 5B). The layered heterostructures containing graphene oxide nanolayers and 20–35 nm bimetallic layers can be easily separated from the silicon matrix by acoustic degradation. The separated layered heterostructures are allowed to spontaneously assemble into micrometer-sized coils through a combination of material stress and weak binding interactions between the graphene oxide layers. During the fabrication process, the diameter of the coils can be tuned not only by varying the thickness of the metal film, but also by choosing the appropriate materials to control the stresses during the coiling process and the function of the structures. Using the same design principle, Hong et al. [157] fabricated tissue cells from banana and fruit extracts onto which a thin layer of platinum was deposited by physical vapor deposition. Spontaneously formed microcoils of highly uniform size were obtained by sonic degradation of the mixed cell–platinum coating matrix in water. The tubular micro-engine prepared by the fruit cell film-assisted deposition method has the ability to move at high speeds in hydrogen peroxide solution by forming a bubble recoil mechanism.

To further improve the kinematic performance of the microtubes, Magdanz et al. [158] designed flexible thermo-responsive micro-nozzles based on the fact that polymers have a reversible folding/unfolding function as they rise and fall with temperature. It is formed by folding polymer layers containing a thin film of platinum. Self-activation in hydrogen peroxide solution is achieved by using platinum as a catalyst. This micro-nozzle enables reversible self-folding in a precise manner by changing the temperature of the solution in which it is placed, and the radius can be adjusted to achieve multiple rapid starts and stops of the micro-nozzle.

#### 2.3.2. Self-Scrolling Technique for Helical Micromotors

Bell et al. [159] were the first to fabricate helical nanomotors using the self-rolling technique. Relying on the tension of a thin layer of material, the rolling technique can transform a straight ribbon structure into a magnetically driven helical structure. For example, Zhang et al. [97] fabricated helical nanomotors using the conventional method of thin film deposition (Figure 5C). The deposited metal layers were first converted into straight ribbons by reactive ion etching, followed by chemical vapor deposition to prepare magnetic heads for magnetic actuation. Due to the presence of internal pressure in the material, the straight ribbon material will form a helical structure by self-rolling when released from the substrate. Typically, the parameters of the helical material can be adjusted by varying the deposition conditions, such as the film thickness, the width of the ribbons and the relative orientation of the ribbons with respect to the crystalline structure of the metal. To further investigate their kinematic properties, Li et al. [160] studied the effect of the magnetic head size of the prepared helical structures on their travelling speed. At lower frequencies, the helical material with a smaller magnetic head moves faster than the helical material with a larger magnetic head due to less viscous drag in the fluid. However, because the larger head contains more nickel magnetic material, it is subjected to a stronger magnetic moment, causing the maximum speed to increase.

In addition to the use of rotating magnetic fields, it is also possible to use electro-osmotic forces generated by electric fields to drive helical substances prepared using thin film deposition [98]. The principle of electro-osmotic actuation is realized using the interface between the surface of the helical structure and the fluid solution. In the presence of an applied field, the stern layer of the helical structure flows, creating a hydrodynamic pressure on the surface of the helical substance that drives the helical structure in the opposite direction. This electro-osmotic actuation of the helical material gives it higher speeds and maneuverability compared to a rotating magnetic field.

The convolution process is founded on stress modulation, facilitating the development and displacement of micro- and nanostructures via the introduction of a prearranged stress distribution within the material. Utilizing the convolution process to prepare micro- and nanorobots presents several clear-cut benefits. Firstly, this approach allows for an extremely flexible structural design and control, facilitating the precise manipulation and motion of micro- and nanorobots through parameter adjustment, including stress distribution and shape modifications. Secondly, the convolution process can be used to produce highly versatile micro-nanorobots that have a range of functions, including motion, deformation and sensing capabilities, achieved through appropriate stress distribution design. Moreover, the convolution process exhibits high scalability and is adaptable to various material systems, including metals, polymers and composites, among others. As a result, it possesses significant potential for a wide range of applications. Nevertheless, the current convolution process technologies for preparing micro- and nanorobots have certain drawbacks and limitations. Firstly, the preparation of the convolution process is quite intricate and necessitates the accurate control of the stress distribution and shape of the material. Secondly, the convolution process might be confined by the mechanical properties and stability of the material, and particular stress distributions could result in the rupture or failure of the material. Furthermore, advancements are required in the preparation technology of the convolution process in order to enhance the accuracy and dependability of preparation. The restrictions in the present technology are largely associated with the precise control and scalability of stress design. In relation to precise control, more intricate stress design methods and process parameters are necessary for achieving more complex micro- and nanorobotic structures and movements. In terms of scalability, more stress design preparation methods for more material systems need to be investigated to meet different application requirements.

### 2.4. Three-Dimensional Laser Writing

#### 2.4.1. Three-Dimensional Printing

**Figure 6:**
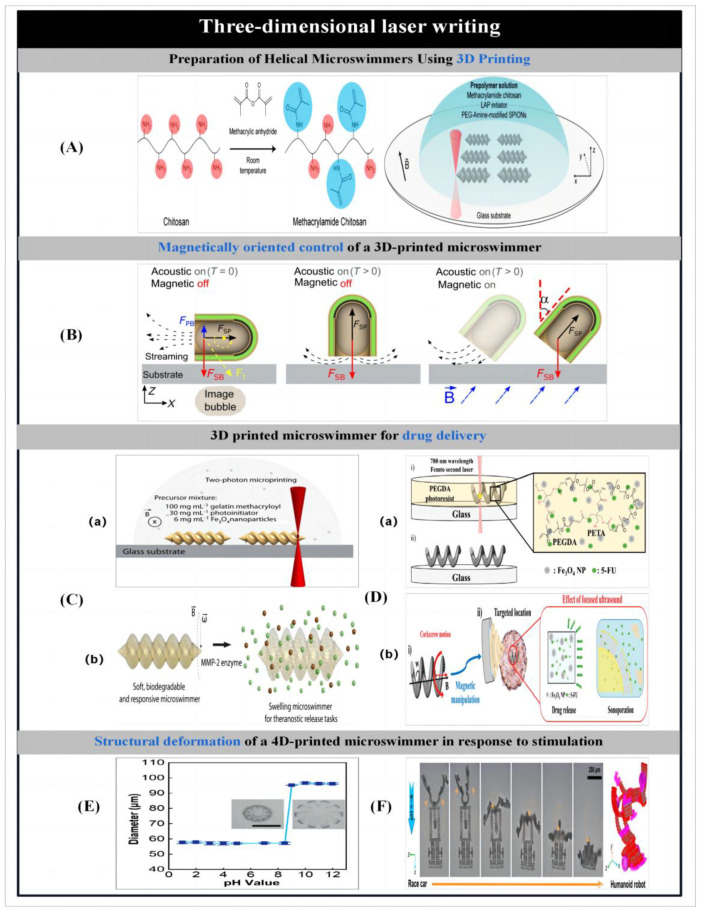
Micromotors fabricated by 3D direct laser writing. (**A**) Preparation of microswimmers with double helical structures using 3D printing of natural derivatives of chitosan. Reproduced from Ref. [20]. Copyright 2018, the American Chemical Society. (**B**) Changes in the motor behavior of acoustically driven microswimmers before and after the application of a magnetic field. Reproduced from Ref. [8]. Copyright 2018, the American Association for the Advancement of Science. (**C**) (a) microswimmers with optimal 3D double-helix structures are prepared using two-photon polymerization by maintaining arrays of nanoparticles with a continuous magnetic field during fabrication; (b) the spiral microswimmer reaches the target location under the action of a rotating magnetic field and subsequently expands and releases the drug under the stimulating action of lysosomal enzymes. Reproduced from Ref. [29]. Copyright 2022, the American Chemical Society. (**D**) (a) preparation of PDM using 3D printing technology; (i) exposure of the TPP laser on the PEGDA photoresist and (ii) the PDM structure after development (b) in the presence of ultrasound, PDM releases drugs in different patterns. (i) locomotion to a targeted area using a RMF and (ii) exposure of the focused ultrasonic beam to the PDM at the targeted area. Reproduced from Ref. [3]. Copyright 2020, Wiley-VCH. (**E**) Effect of pH change on microsphere diameter. Reproduced from Ref. [170]. Copyright 2019, Elsevier Ltd. (**F**) Completion of 3D–3D (car–humanoid robot) shape transformation by microstructures in an acidic environment. Reproduced from Ref. [173]. Copyright 2020, the American Association for the Advancement of Science.

At the micro- and nanoscale, 3D direct laser writing (DLW) allows the precise batch preparation of complex polymer structures with high resolution [161,162,163]. Typically, we use DLW techniques to fabricate helically structured micromotors (Figure 6A) [9,20,31,65]. For example, Tottori et al. [99] first fabricated magnetic helical nanomotors by depositing negative photoresist on a substrate and then using DLW. When a laser beam is focused on the photoresist, two-photon polymerization occurs at the focal point of the laser beam. The unused photoresist is then removed to expose the helical polymerized structure. Finally, a layer of nickel and titanium is deposited on the surface of the helical structure using an electron beam evaporation technique to make it bioadaptable and magnetic for magnetic actuation. In contrast to the manufacture of helical motors using negative photoresists, the DLW technique allows the fabrication of templates containing 3D cavities which are then filled by electrodeposition using positive photoresists. The combination of electrodeposition and DLW techniques helps to prepare a wide range of materials into complex 3D structures. For example, Ren et al. [8] used electrodeposition and DLW to fabricate bubble-based ultrasound-driven microswimmers that are not only able to move autonomously in three-dimensional space, but can also selectively transport individual composite colloids as well as mammalian cells in relatively dense populations without affecting the surrounding materials. In a MHz acoustic field, the microswimmer is subject to two forces: the secondary Buell–König force and the locally generated acoustic current driving force. The combination of these two forces allows the microswimmer to move freely at three-dimensional boundaries or in free space in the presence of a magnetic field (Figure 6B). Similarly, Zeeshan et al. [100] fabricated helical micromotors consisting of a ferromagnetic alloy head and a helical polymer tail using a template-assisted two-step electrodeposition process. A 3D photoresist template was prepared according to DLW to provide a mask for the electrodeposition process, a 3D microcavity was fabricated using orthochromatic photoresist, and the cobalt–nickel alloy and polymer were filled into the microcavity by electrodeposition. The magnetic head and polymer tail are used for magnetic manipulation and liquid actuation, respectively.

In order to make the prepared magnetic helical microrobots biocompatible for better application in living organisms, Ceylan et al. [29] achieved 3D printed optimal microswimmers with double helical structures by two-photon polymerization of magnetic precursor suspensions using methacryloyl gelatin and superparamagnetic Fe_3_O_4_ nanoparticles (Figure 6C-a). The microswimmers can take up loads and swim under the action of a rotating magnetic field. At normal physiological concentrations, the microswimmer can be biodegraded by metalloproteinase-2 (MMP-2) in the body and dissolved into non-toxic substances within 118 h. The microswimmer can respond to its dissolving enzyme lesion by swelling, which then increases the release of the implant loading molecule (Figure 6C-b). Similarly, Park et al. [3] reported a magnetically driven, porous and degradable microrobot (PDM) that can enhance the therapeutic effect on cancer cells by increasing the drug concentration and porous effect. The PDM is composed of a helical flexible polymer chassis by 3D printing a substrate containing magnetic nanoparticles and the anticancer drug 5-fluorouracil (Figure 6D-a). The Fe_3_O_4_ nanoparticles encapsulated within the microrobot in the presence of an applied rotating magnetic field enabled the precise wireless control of the PDM. The porous PDM not only facilitates the increase in its surface area for drug loading, but the formation of cavities makes it highly responsive to external acoustic stimuli for drug release. Depending on the conditions of ultrasound exposure, the drug released from the PDM can be released in one of three modes: natural, burst and constant, according to the command (Figure 6D-b). The results of in vitro tests showed that different release modes had different therapeutic effects. Among them, the burst and constant modes have the best therapeutic effect, in which the activity of cancer cells is drastically reduced under these two drug release modes.

In addition to the preparation of helical structures, DLW can be used to prepare a variety of microstructures by controlling the geometry of the sample. Kim et al. [164] used DLW to prepare a multifunctional microrobot for targeted cell delivery. To make the microrobot magnetic and bioadaptable, the surface of the microrobot was coated with a nickel and titanium layer, and its three-dimensional porous structure could be used for cell culture. Because the materials that make up the three-dimensional structure are not cytotoxic to myoblasts, the structure allows cells to adhere, engraft and proliferate on it. The multifunctional microrobot can be targeted for delivery in the presence of an applied magnetic field. Similarly, Kümmel et al. [93] used DLW to fabricate light-driven asymmetric L-shaped microswimmers. First, L-shaped structures were fabricated from negative photoresists by photolithography. Gold was then deposited on their surface by thermal evaporation. During the evaporation process, the circular wafer to which the particles were attached was tilted at an angle so that deposition occurred at the front end of the short arm. Finally, the L-type particles were released from the substrate using acoustic degradation by suspending them in a homogeneous mixture of water and 2,6-dimethylpyridine at a critical concentration. The metal layer was heated under light conditions, causing local delamination of the solvent and allowing the particles to achieve autoelectrophoretic motion. With the variety of micromotor shapes, Ten et al. [165] introduced the motion of asymmetric colloidal particles with uniform mass density and well-defined shapes. It was found that shape anisotropy alone was sufficient to induce gravitational motion, i.e., a preferential up and down motion. Such shape-anisotropic particles can deflect light, which does not carry angular momentum, and thus generate the momentum required for rotational motion. We are now increasingly using 3D printing to fabricate complex light-driven rotors that can be manipulated with optical tweezers.

#### 2.4.2. Four-Dimensional Printing

Soft materials can quickly respond to external stimuli and change shape in three dimensions, so they have been widely used in 3D printing technology to prepare microrobots [166,167]. Cvetkovic et al. [168] introduced 3D-printed hydrogel-based bio-robots [169,170]. The microrobot has an asymmetric physical design in which electrical stimulation induces cell contraction in muscle strips to achieve reticular motion driven by skeletal muscle strips. Meanwhile, Yuk et al. [171] presented a high performance 3D-printable conductive polymer ink based on poly-3,4-ethylenedioxythiophene and poly(ethylene styrene sulfonate) (PEDOT: PSS) for 3D printing conductive polymers, resulting in miniature conductive polymers with ultra-high printability, easy preparation, high resolution and a high aspect ratio. This 3D-printed conductive polymer can be transformed into highly conductive and soft hydrogel microstructures. The microrobots produced by this 3D printing technique can be shape-shifted in response to external stimuli, so we also refer to this fabrication technique as 4D printing.

This 4D printing technique allows 3D structures to reversibly change shape in response to external stimuli (Figure 6E) [170]. Taking advantage of the deformability of liquid crystal elastomers, Ambulo et al. [117] printed thermo-responsive liquid crystal elastomers (LCE) in 3D structures with controlled molecular order. Under the influence of heat, the aligned LCE filament reversibly shrinks by 40% along the printing direction. In addition, origami, another deformable material, has been widely used to fabricate microrobots. Ge et al. [172] used 4D printing to design and fabricate active origami that can be automatically folded from flat paper into parts with complex 3D shapes. The origami folding mode was achieved by precisely printing shape memory polymer fibers onto an elastic matrix, which was used as an intelligent active hinge. Subsequently, to achieve more complex deformation behaviors, Huang et al. [173] constructed 3D reconfigurable microstructures using programmable modular design principles. First, hydrogels containing two-photon polymers with stimulus-responsive mechanisms were prepared to construct 4D micromodules via 4D printing. Subsequently, reconfigurable microstructures capable of complex 3D–3D shape transitions were constructed by assembling the 4D micromodules. The Denavit–Hartenberg (DH) robotic arm dynamics parameter was used to guide the assembly of the micromodules and the motion of the assembled chain blocks. Finally, the DH parameter was used to guide the motion of the assembled components to complete the shape transformation of the microstructure (vehicle–humanoid robot) (Figure 6F). Subsequently, in order to apply the deformable behavior of the microrobot in living organisms, Scarpa et al. [174] prepared 3D pH-responsive microstructures with high molecular weight poly(ethylene glycol) diacrylates (PEG-DAs) via DLW, which allowed them to become structural and functional components for pH sensing in soft biological tissues. The microstructures, which come in two different shapes, pyramid and dome, are extremely fast at sensing pH at the microscale, typically swelling and deforming in response to pH stimuli within 15 min. This result provides a pH-responsive tool for biosensing of cells and biological tissues.

The technique of 3D printing is popular for creating micro and nano robots. This method of preparation offers many benefits for manufacturing micro- and nanorobots. It involves stacking layers of materials using a print head, which is controlled to prepare the structures precisely. First, 3D printing provides a high level of design flexibility, allowing for intricate control of micro- and nanorobot structures and morphology. This leads to greater adaptability in functionality and performance. Second, the production process enables personalized and customized design, manufacturing and preparation to meet specific requirements and application scenarios. Furthermore, 3D printing exhibits high scalability and reproducibility making it an appropriate technology for large-scale production of micro- and nanorobots consequently enhancing preparation efficiency and reducing costs. Nevertheless, the current 3D printing technologies for micro- and nanorobot preparation have some drawbacks and constraints. Firstly, the resolution and preparation accuracy are restricted, rendering it impossible to achieve fine structure preparation at the nanoscale. Secondly, the properties of the material, including its fluidity and viscosity during the printing process, may result in surface roughness and morphological errors in the micro-nanorobots that are prepared. Furthermore, 3D printing is a relatively slow process, and longer printing times may result in material instability and quality changes. The present technology is restricted primarily by the selection of materials, nanoscale control and multi-material integration. Further development of nanomaterials suitable for 3D printing is required to address their rheological properties and printability. Additionally, enhancing the resolution and precision of 3D printing is crucial to achieve finer micro- and nanostructure preparation at the nanoscale. Multi-material integration poses a challenge, and compatibility and interfacial connection of varying materials must also be considered.

### 2.5. Assembly of Materials

Self-assembly is a spontaneous reorganization process that can assemble disordered component systems into ordered structures. The self-assembly process is usually reversible and the structures formed by self-assembly are held together by non-covalent interactions. The building blocks of self-assembly are not limited to atoms and molecules, but include nanostructures of various compositions. Thus, the unique properties of self-assembled monolayers can be exploited to prepare a variety of microrobots. Complex three-dimensional structures can be obtained by assembling self-assembled monolayers. In addition to using the self-assembly of materials to create the desired structure, it is also possible to encapsulate or incorporate the desired component into another material so that the desired component is contained within the microrobot.

#### 2.5.1. Layer-by-Layer Assembly

Layer-by-layer self-assembly (LbL) is a nanofabrication technique for forming multilayer films by depositing alternating layers of oppositely charged materials. It is a relatively simple method that can incorporate a wide range of versatile materials such as small organic molecules, inorganic compounds, macromolecules and colloids, and can be applied not only to readily accessible solvent surfaces, but also to a wide range of different templates. Typically, nanoparticles with catalytic properties are added to the assembled multilayer structure so that the particles can move in a hydrogen peroxide solution.

**Figure 7:**
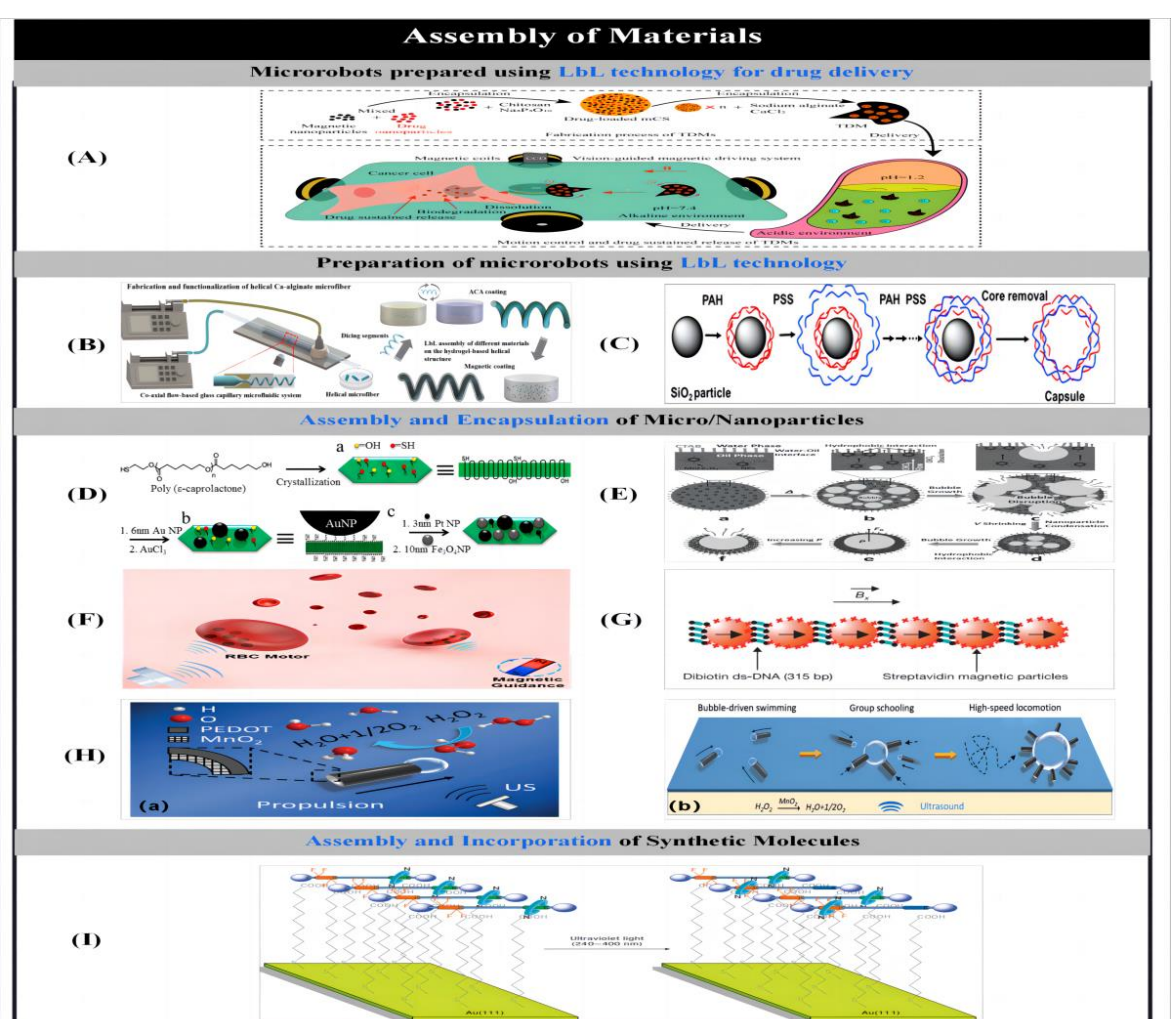
Fabrication of micromotors by assembly of materials. (**A**) Preparation of TDMs, exercise, and the process of drug release under alkaline stimulation. Reproduced from Ref. [63]. Copyright 2020, Elsevier Ltd. (**B**) Combination of microfluidic synthesis, polyelectrolyte complexation and surface-coated magnetic nanomaterials for the preparation of magnetic microrobots with multilayered capsule helical structures. Reproduced from Ref. [57]. Copyright 2021, the Royal Society of Chemistry. (**C**) Fabrication of (PSS/PAH)5 hollow capsules through LbL assembly. Reproduced from Ref. [74]. Copyright 2012, the American Chemical Society. (**D**) Synthetic procedure of a nanoparticle-based PSC nanomotor. (a) PSC of R-hydroxyl-ω-thiol-terminated polycaprolactone (HO PCL SH) 40 was grown using a self-seeding method.(b) a AuNP was first immobilized onto the PSC surface through its interaction with thiol groups. (c) Considering that the surface SH groups under AuNPs react with gold atoms to form S Au bonds.Reproduced from Ref. [104]. Copyright 2013, the American Chemical Society. (**E**) Potted micromotors obtained by self-assembly of hydrophobic MnFe_2_O_4_@OANPs in volatile oil droplets by growing bubble templates. Reproduced from Ref. [12]. Copyright 2015. Wiley-VCH. (**F**) Schematic of magnetically guided, ultrasound-propelled red blood cell micromotors in whole blood. Reproduced from Ref. [105]. Copyright 2014, the American Chemical Society. (**G**) Schematic illustration of a chain of magnetic particles linked by DNA. Reproduced from Ref. [156]. Copyright 2005, Nature Publishing Group. (**H**) (a) oxygen bubbles generated by the degradation of hydrogen peroxide fuel by a micromotor composed of PEDOT/MnO_2_ oscillate under the effect of ultrasound, which pushes the micromotor to move; (b) bubble oscillations in the ultrasound field cause individual micromotors to aggregate, forming dandelion-like micro-clusters. Reproduced from Ref. [6]. Copyright 2020, Wiley-VCH. (**I**) Photo-responsive surface based on switchable fluorinated molecular shuttles to expose or conceal fluoroalkane region (orange). Reproduced from Ref. [176]. Copyright 2005, Nature Publishing Group.

Wu et al. [102] used layer-by-layer deposition instead of electrodeposition to fabricate microtubular motors after thin film template-assisted electrodeposition. First, two oppositely charged materials, chitosan and sodium alginate, were alternately deposited on the nanoporous film, followed by the assembly of platinum nanoparticles into the film nanopores, and when the film was dissolved, the nanotubes with catalytic platinum layers on the inner wall could be obtained. Such nanotubes formed by LbL can be used not only as automated-type motors, but also as smart payloads. Nanotubes are capable of drug loading, targeted delivery and remote release close to tissues and cells in the living body. However, when targeted drug delivery is actually performed, the acidic conditions caused by tumors make the drug-loaded microtubes less therapeutically effective. To solve this problem, Wu et al. [63] designed bilayer drug-loaded microrobots (TDMs) with an outer layer of calcium alginate hydrogel and an inner layer of magnetic chitosan microspheres (mCSs). The TDMs delivered the drug-loaded mCSs to a predetermined location along a desired route under the action of a visually guided magnetic actuation system. Under acidic conditions, the outer layer of the TDMs was able to protect the mCSs, whereas under alkaline conditions, the calcium alginate hydrogel dissolved at a suitable rate, allowing the continuous release of the mCSs (Figure 7A). Subsequently, to further improve the efficiency of targeted drug delivery, Liu et al. [57] prepared a magnetic microrobot with a multilayer capsule helical structure by combining microfluidic synthesis, polyelectrolyte complexation and surface-coated magnetic nanomaterials. The microrobot not only has a superior loading capacity, but can also release the capsule in a controllable manner (Figure 7B). The microrobot’s helical structure was first fabricated from calcium alginate microfibers using a coaxial capillary microfluidic system. It was then wrapped with a polyelectrolyte complex membrane and its surface modified with magnetic nanoparticles. After assembling the multifunctional units in stepwise layers, the helical structure is transformed into a helical capsule. The helical microrobot achieves rotational motion under the action of a 6-degrees-of-freedom electromagnetic drive system with different frequencies. The capsule also responds to ionic stimulation through the presence of a semi-permeable membrane that allows the controlled release of a trigger load in response to ionic stimulation.

In addition to tubular and helical micromotors, the LbL technique can also be used to fabricate Janus micromotors. For example, Wu et al. [74] fabricated Janus capsule motors partially coated with platinum nanoparticles using a combination of template-assisted hierarchical assembly and microprinting (Figure 7C). First, five layers of oppositely charged polystyrene sulfonate (PSS)/polyallylammonium hydrochloride (PAH) were deposited on the surface of the silica template and then physically contacted on the top of the substrate with a stamp loaded with platinum particle ink. The subsequent removal of the silica template results in a hollow capsule partially coated with platinum particles. The asymmetric movement of the platinum particles and the hollow structure enable the generation of directional forces and encapsulation of the drug. Loading and release of the drug can be achieved by adjusting the permeability of the capsule using an organic solvent, and can be magnetically controlled by applying a magnetic material to the wall of the capsule.

#### 2.5.2. Assembly and Encapsulation of Micro/Nanoparticles

Another approach used to prepare Janus micromotors is to utilise the asymmetric assembly of nanoparticles, which provides the required directional thrust for the motion. Dong et al. [104] fabricated Janus micromotors by directional self-assembly of nanoparticles on a single-crystal polymer (PSC) of (HO-PCL-SH) (Figure 7D). Due to the presence of a large number of thiols and hydroxyl groups on the surface of the single-crystal polymer, metal nanoparticles can be implanted into the single-crystal polymer by exploiting the interactions between gold, Fe_3_O_4_ and platinum nanoparticles and functional groups. Platinum nanoparticles can degrade the hydrogen peroxide solution and make the single crystal polymer flow in solution. Gold and Fe_3_O_4_ particles can be made to move directionally in the presence of a magnetic field. The polymerized oral cells are nano-sized bowl-like structures formed by controlled deformation of the polymerized vesicles. The stable nanocavity and tight opening and closing control can act as a physical trap for nanoparticles. When nanoparticles are active, they can also transform the oral cellular morphology into nanoreactors. The self-assembly technique can be used to prepare not only common regular-structured microrobots, but also non-regular-structured microrobots. Mou et al. [12] prepared micromotors consisting of pot-shaped hollow manganese ferrite particles. They demonstrated magnetic modulation of the micromotors and assembly of the particles using a growth bubble template. In an oil droplet consisting of trichloromethane and hexane, nanoparticles consisting of hydrophobic manganese ferrite with oleic acid were assembled to form a compact particle shell layer due to the hydrophobic interactions between the particle surfaces. As the encapsulated oil droplets are continuously vaporized to form high pressure bubbles, the compact shell layer ruptures. This creates a single hole in the shell, resulting in an asymmetric jug-shaped micromotor (Figure 7E).

In addition, the assembly of microrobots can also be achieved by exploiting the throughput and adsorption properties of biological cells. Wilson et al. [39] selectively trapped catalytically active platinum particles in the nanocavity of oral cells. The oral cells were engineered to catalyze the decomposition of hydrogen peroxide to form a rapid discharge, which induced the generation of propulsive force to achieve automated locomotion. In addition to the assembly of platinum nanoparticles into micromotors, Wu et al. [105] demonstrated the direct conversion of red blood cells into functional micromotors, enabling the micromotors to function during acoustic actuation and magnetic guidance. By loading Fe_3_O_4_ nanoparticles into hemoglobin cells, the asymmetric distribution of Fe_3_O_4_ particles within the cell creates a net magnetization. This results in the magnetic alignment and guidance of the micromotor when driven by acoustic waves (Figure 7F). In addition, the adsorptive properties of biological cells can also be exploited for particle assembly: Dreyfus et al. [103] introduced a linear chain of colloidal magnetic particles which, as a flexible artificial flagellum, is attached to blood erythrocytes by DNA attachment. The flagellum would be aligned with an external homogeneous magnetic field and driven by the oscillating action of the transverse magnetic field. The drive induces a beating pattern in the artificial flagellum, resulting in movement of the entire structure. The speed and direction of movement can be controlled by adjusting the magnetic field parameters (Figure 7G).

In addition to building microbots by assembling nanoparticles, it is also possible to assemble prepared particles into other structures in response to external stimuli. For example, Palacci et al. [106] synthesized photoactivated colloidal particles in suspension by non-equilibrium forces. The particles can form, break, explode and reorganize. The particles are induced to produce osmotic and light-transmissive effects by light-activated particles. The excitation and coupling of the self and mutual attraction effects of the particles allowed the particles to assemble into two-dimensional active crystals. Subsequently, Lu et al. [6] described a method for the rapid growth and movement of dandelion-like microclusters assembled from catalytic tubular microengines. The self-generated bubbles of the microtubes oscillate under the action of ultrasonic waves, allowing the microcluster to overcome the resistance created by a large and disordered number of bubbles within a single cell (Figure 7H-a). Driven by ultrasonic waves, individual tubular manganese dioxide micromolecules float rapidly in a surfactant-free hydrogen peroxide solution, led by self-generated oxygen bubbles. The large bubble nucleus formed by the fusion of multiple microbubbles was excited and oscillated by the acoustic wave, and the resulting locally enhanced acoustic field attracted more individual micromotors to cluster around it, creating dandelion-like micro-clusters (Figure 7H-b).

#### 2.5.3. Assembly and Incorporation of Synthetic Molecules

In addition to using the self-assembly of materials to build the desired structure, it is also possible to make the desired component contained in a microrobot by incorporating it into another material [175]. Vicario et al. [111] incorporated manganese peroxidase mimicry into a molecular system by covalent conjugation to allow it to convert chemical energy for actuation. Zhang et al. [112] used artificial molecules for the assembly and synergism to prepare an automated motor micromotor. The micromotors were powered by a rapid degradation reaction of poly(2-ethylcyanoa-crylate) (PECA). Micron-sized anion exchangers with PECA adsorbed on the porous structure are semi-coated with poly(methyl methacrylate), and OH^—^ released during ion exchange induces degradation of PECA at the uncoated end. The degradation process produces ethanol and the resulting ethanol is capable of creating a surface tension gradient to push the particles forward.

In the development of light-driven motors, it is often possible to modify the surface of particles with photoisomerized molecules of azobenzene. The photoisomerized molecules create an interfacial tension gradient when irradiated with asymmetric light, resulting in net mass transfer between microdroplets and nanoparticles. Abid et al. [107] described the motion of polymer nanoparticles coated with azobenzene 16 nm in diameter. The dye grafted onto the nanoparticles can act as a molecular driver and its photoisomerization can provide sufficient mechanical energy to propel the particles. It allows the particles to move straight through the aqueous medium to the region of lowest intensity at 15 um/s. Based on the reversible cis-trans isomerization of the azobenzene molecule, it is possible to reversibly control the light. The directed movement of droplets modified with azobenzene molecules on the substrate surface was also achieved [108]. Similarly, Berna et al. [176] described the directed movement of miniature droplets on a light-responsive surface consisting of a monolayer of rotaxane molecular shuttles (Figure 7I). The millimeter-scale directional transport of droplets on the surface was achieved by using stimuli responsive to the Brownian motion of the rotaxane, exposing or concealing halothane residues, thereby changing the surface tension.

Another application of light-driven micromotors is to allow micromotors to induce controlled motion in large objects by implanting them in liquid crystal elastomer films. For example, Vicario et al. [109] introduced molecular motors characterized by a right-handed helical structure. The rotor site controls the direction of rotation of the motor and there is a single three-dimensional center at the rotor site, with a carbon–carbon double bond in the central structure acting as an axis. The micromotor induces the rotation of the microrobot on the liquid crystal film in the presence of light. Subsequently, Camacho-Lopez et al. [110] dissolved nitrogen-containing dyes instead of covalently bonding them to the LCE, whose mechanical deformation becomes very pronounced when irradiated with non-uniform visible light. This light-induced rapid deformation allows the LCE to interact with its environment in a novel way. When a liquid crystal elastomer with a reference dye floating on the surface of water is irradiated with light from above, it exhibits an off-axis behavior.

Material assembly is based on assembling micro- and nano-components of different materials together for the construction and functional realization of micro- and nano robots. Material assembly for the preparation of micro- and nanorobots has some significant advantages. Firstly, it can achieve multi-material integration, and the versatility and complexity of the micro-nanorobots can be realized by assembling different components. Secondly, material assembly is highly scalable and can adapt to the needs of micro- and nanorobot preparation at different scales and different material systems. In addition, material assembly also has high preparation efficiency, which can realize the needs of mass production and large-scale preparation of micro- and nanorobots. However, there are some drawbacks and limitations of the current technology for the preparation of micro- and nanorobots by material assembly. Firstly, the precision and controllability of material assembly are limited, and fine assembly at the nanoscale cannot be achieved. The accuracy and consistency of the assembly may be affected due to the variations in the size and shape of the micro- and nano-scale components. Secondly, the process complexity of material assembly is high, requiring precise control of the position and connection between components, which is demanding in terms of process and equipment. In addition, the scalability of material assembly is challenging, especially when preparing and integrating multiple components on a large scale, which may face problems in process control and inter-component interactions.

### 2.6. Biohybrid Technique

Natural systems themselves can provide a variety of microscale motors such as F_1_-adenosine triphosphate synthase, kinesin, myosin, etc. These natural micromotors can convert chemical energy in the surrounding medium into mechanical energy for their own movement. In addition, mobile units such as bacteria and muscle joints can also achieve self-powered motion in low Reynolds number fluids. Combining the biological properties of natural motors with man-made molecules opens up new possibilities for the fabrication of micro- and nanorobots. This section describes the fabrication of micro- and nanorobots using single biomolecules as well as micro-units.

#### 2.6.1. Use of Biological Molecules

**Figure 8:**
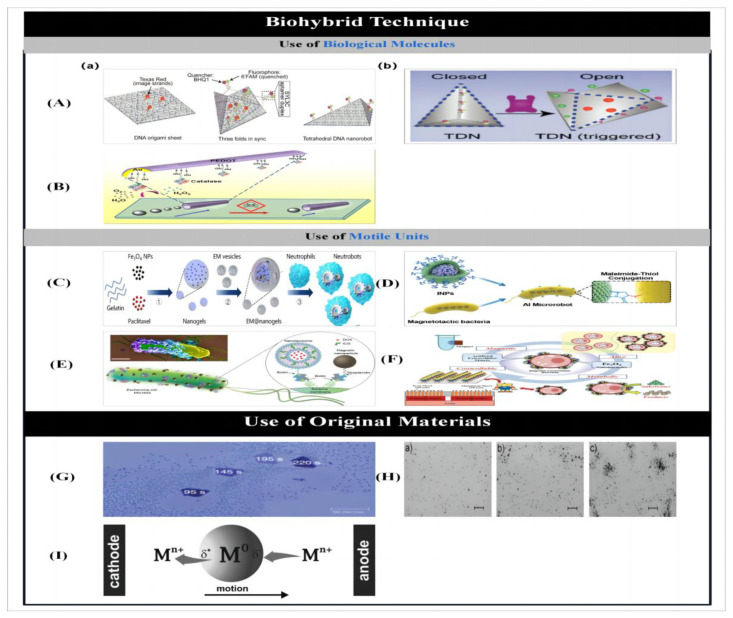
Fabrication of micromotors by biohybrid techniques and use of original materials. (**A**) (a) preparation process of TDA; (b) EpCAM induces conformational changes in TDA to recognize cancer cells. Reproduced from Ref. [34]. Copyright 2021, the Royal Society of Chemistry. (**B**) Use of biocatalytic microjets with enzyme immobilized at the inner gold surface as microfish for testing water quality. Reproduced from Ref. [75]. Copyright 2012, the American Chemical Society. (**C**) Preparation of neutrobot. Reproduced from Ref. [32]. Copyright 2021, the American Association for the Advancement of Science. (**D**) Preparation of an AI microrobot with paramagnetic/conducting capability. Reproduced from Ref. [25]. Copyright 2020, Wiley-VCH. (**E**) Integration of magnetic nanoparticles and nanoliposomes loaded with photothermite and chemotherapeutic molecules onto Escherichia coli for biohybrid microbots. Reproduced from Ref. [180]. Copyright 2022, the American Association for the Advancement of Science. (**F**) Preparation and movement of alg-mag-cells and the cell culture process. Reproduced from Ref. [15]. Copyright 2016, Wiley-VCH. (**G**) Movement of a TiO_2_ particle in a suspension of SiO_2_ particles under UV. Reproduced from Ref. [76]. Copyright 2010, Wiley-VCH. (**H**) AgCl particles in deionized water (a) before UV illumination; (b) after 30 s; and (c) after 90 s of UV exposure.Scale bars: 20 mm. Reproduced from Ref. [90]. Copyright 2009, Wiley-VCH. (**I**) Scheme illustrating principle of dynamic bipolar self-regeneration. Reproduced from Ref. [88]. Copyright 2010, the American Chemical Society.

Biomolecules can be assembled with engineered components to form hybrid microsystems. Song et al. [17] constructed a targeted drug-delivery microrobot for cancer cell therapy. The carboxyl groups on the surface of the three-microbead microbots were first linked by strong covalent bonds, followed by the adhesion of the azobenzene photothermally responsive material on the surface of the carboxyl groups. The microbots were not only magnetic, but also remained very cytocompatible at higher concentrations (200 μg/mL). Once the microbots reach the target site under the influence of a magnetic field, the drug is released under the photothermal effect induced by near-infrared light. In addition, hybrid nanosystems can also be prepared by incorporating DNA into artificial components. Liu et al. [34] described a stable DNA nanorobot (TDA) (Figure 8A-a). The TDA was able to undergo controlled conformational changes in response to stimulation of epithelial cell adhesion molecules specifically expressed in the tumor cell cycle. Although the TDA undergoes local deformation during the folding process, the overall structure remains highly stable. Compared to traditional targeted drug-delivery particles, TDA has lower cytotoxicity and target specificity. Therefore, TDA is not only effective against nuclease catalysis, but also has the ability to precisely target EpCAM-positive cells (Figure 8A-b). On this basis, Sengupta et al. [177] introduced an enzyme that is not dependent on the surface immobilization of adenosine triphosphate. It can act as a self-driven micropump in the substrate. This surface-immobilized enzyme (catalase, lipase, etc.) can undergo a catalytic reaction that is self-driven by the fluid density gradient generated by the reaction process. The speed of the micropump increases with increasing the substrate concentration and reaction rate.

To enable better in vivo applications for microrobots, Sanchez et al. [113] constructed a self-powered hybrid micro-engine by incorporating catalase in a covalent form into the ventral lumen of a coiled microtubule. Positively generated bubbles generate drag forces to rotate them, allowing them to move in low concentrations of hydrogen peroxide fuel. The enzyme that catalyzes the breakdown of the peroxidase is inhibited by contaminants in the water solution. Taking advantage of this property, Orozco et al. [75] introduced a novel micromotor-based water quality testing strategy. This strategy is based on changes in the driving behavior of the artificial biocatalytic microswimmer in the presence of aquatic pollutants (Figure 8B).

#### 2.6.2. Use of Motile Units

It is often difficult to use biological motors outside the cell, so intact motile cells are used as actuators for microsystems. Zhang et al. [32] were the first to use intact neutrophils to construct a neutrobot (Figure 8C). Neutrophils constructed the neutrobot by phagocytosing *E. coli*-coated, drug-loaded magnetic nanogels. The *E. coli* film wrapping not only enhanced the phagocytosis efficiency, but also prevented drug leakage inside the neutrophils. In the presence of a rotating magnetic field, the neutrobot exhibits controlled locomotion within the vasculature and actively accumulates in the brain. The neutrobot then crosses the blood–brain barrier and, by chemotaxis through a gradient of inflammatory factors, reaches the tumor cells and releases the drug to inhibit tumor cell growth. On the same principle, Al-Fandi et al. [33] reported a novel method for self-detection of tumor cells using living nanorobots. The living nanorobot is a non-pathogenic strain of *E. coli* equipped with a naturally synthesized biosensor system. The nanosensing system has an affinity for the angiogenic factor VEGF, which is over-expressed by cancer cells. Therefore, selective targeting of tumors can be achieved by using swimming *E. coli* as a self-navigating drug delivery vehicle.

In addition to achieving movement in response to external stimuli, biohybrid microrobots can also rely on their own deformability to achieve movement. For example, Magdanz et al. [114] presented a miniature bio-based robot consisting of mobile sperm cells and magnetic microtubules. The microrobot does not rely on any toxic fuel and achieves movement solely by driving the cell flagellum. In addition, the magnetic microtubules, which are formed from convoluted thin films, contain lumens that can be used to capture cells and guide sperm cells to move in the presence of an applied magnetic field. Meanwhile, Williams et al. [178] introduced a micro-scale series of biohybrid swimmers composed of polydimethylsiloxane filaments. They have a stiff, short head and a long, thin tail. Actuation is achieved by selectively culturing cardiomyocytes in the tail and using the cardiomyocytes to contract or deform the filaments.

Due to their high biological adaptability, biohybrid microbots have been widely used in biomedical applications [15,25,179,180]. For example, Xing et al. [25] constructed a biological microrobot (AI microrobot) by combining magnetospirillum magneticum (AMB-1) with indocyanine green nanoparticles (Figure 8D). The former enabled the microrobot to move towards the tumor region in response to the internal hypoxia effect and the applied magnetic field. The latter serves as a fluorescent imaging agent and a photothermal therapeutic agent. Under the photothermal effect, the microrobots will continuously migrate to the internal hypoxic region of the tumor, effectively eradicating the solid tumor. Meanwhile, Akolpoglu et al. [180] combined magnetic nanoparticles and nanoliposomes loaded with photothermal agents and chemotherapeutic molecules and integrated them into Escherichia coli with 90% efficiency to form a bacterial biohybrid (Figure 8E). This biohybrid microrobot not only retained its original motility, but was also able to navigate through the biological matrix and colonize the tumor spheroids in the presence of an applied magnetic field. The drug is then released under the stimulation of near-infrared light when it reaches the target site. In addition to targeted drug delivery, biohybrid microrobots can also be used for cell manipulation: Shi et al. [15] applied a monolayer of alginate hydrogel to the periplasm of microbial cells doped with iron tetraoxide nanoparticles. The iron tetraoxide particles were uniformly distributed on the hydrogel shell, resulting in the formation of algal magnetic cells (alg-mag cells), which provide the microbial cells with an artificial extracellular matrix (Figure 8F).

Biohybridization is an innovative technique that combines biological tissues with inorganic or organic materials to create micro- and nanorobots. This process offers numerous advantages for the construction and functional realization of such robots. It is a promising approach that has recently gained traction in the field of robotics. Firstly, biohybridization can enable micro- and nanorobots to achieve highly controllable and self-adaptive performance utilizing the self-assembly and self-repair mechanisms present in living organisms. Secondly, biohybridization can combine the beneficial properties of both biomaterials and inorganic/organic materials, resulting in enhanced functional characteristics and enabling a wider range of micro- and nanorobot functions. Furthermore, biohybridization possesses favorable traits of biocompatibility and biodegradability, thus aiding in the introduction and gradual disappearance of micro- and nanorobots in living organisms. However, the present technology for preparing micro- and nanorobots via biohybridization poses several limitations and drawbacks. The initial phase of biohybridization necessitates meticulous control over the interaction and assemblage process between the material and the organism. Due to the intricate internal environment of the organism and the specificity of biological tissues, the preparation process presents significant challenges in terms of reproducibility and stability. Secondly, the activity of biological enzymes, immune responses, and other factors can impact the function and stability of micro- and nanorobots produced through biohybridization, thus limiting their performance. Furthermore, the production of micro- and nanorobots through biohybridization is subject to limitations in terms of scale and morphology, hindering the ability to achieve precise nanoscale control and structure. The constraints of current technology are primarily evident in the controllability and stability of biohybridization. Achieving more precise assembly and control necessitates further research and understanding of the interactions between organisms, materials and their regulatory mechanisms. The reliability and durability of micro- and nanorobots produced by biohybridization in complex environments must be assessed to enhance their long-term utility, stability and performance.

### 2.7. Use of Original Materials

In addition to several of the preparation methods mentioned above, some unmodified natural materials can be used directly as microengines. Cyclodextrins (CDs), as an amylase-degradable host molecule, can combine with surfactants, alkanes, alkylamines, fatty alcohols and aromatic compounds to form supramolecular compounds. Xiao et al. [35] constructed enzyme-responsive nanosystems through host–guest interactions, where the complexation of CDs with surfactants formed a variety of nanostructures such as vesicles and microtubules. These supramolecular structures are capable of loading water-soluble molecules or functional nanoparticles and can be released on demand in the presence of α-amylase. In addition to the use of biomolecules, naturally occurring metal oxides can be used to directly construct micromotors. Mushtaq et al. [13] reported the catalytic degradation of organic compounds by exploiting the magneto-electric properties of core-shell nanoparticles composed of cobalt–bismuth ferrite (CFO-CBO). By combining the magnetostrictive properties of CFO with multiferroic BFO, micromotors capable of responding to magnetoelectric stimulation were fabricated. Under the influence of an applied magnetic field, the micromotor is able to break down organic pollutants in an advanced oxidation process, thereby purifying water. No additional sacrificial molecules or catalysts need to be added to the process.

In addition to using the magnetic properties of natural materials and interactions between material molecules to achieve actuation, the natural photoreceptors of natural materials can also be used to drive particle motion. For example, Hong [76] and Ibele [90] et al. fabricated microsystems by exploiting the fact that photoreceptors can generate concentration gradients (TiO_2_ (Figure 8G) and AgCl (Figure 8H)) in the surrounding local environment when stimulated by light. The movement of the microrobots is based on the mechanism of light-induced self-diffusion electrophoresis. Under UV light irradiation, a concentration gradient is created around the particles, causing them to move in the direction of the gradient. Similarly, Wentao et al. [181] perturbed the chemical equilibrium of the silver phosphate particle system itself by adding or removing ammonia. This resulted in a rapid transition between two population behaviors, repulsion and aggregation, of the particles when stimulated by UV light. Based on light-driven micromachines, which usually consist of materials containing metal segments, Villa et al. [28] introduced a metal-free tubular micromotor based on graphitic carbon nitride. Under visible light irradiation, the micromotor achieved motion through a photocatalytically induced bubble recoil mechanism without the need to incorporate metal parts or biomolecules into its structure.

The electrochemical properties of natural materials can also be used to construct micromotors. In general, bipolar electrochemistry enables the actuation of microrobots by inducing asymmetric reactions at the end of the particles. To control the motion of a metallic microrobot, Loget et al. [88] introduced a method based on bipolar electrochemistry using a dynamic bipolar self-regeneration mechanism. First, the metal is deposited on both ends of the substrate by electrodeposition. Then, in the presence of an applied electric field, different redox reactions take place at the two ends of the substrate, where the deposition of metal at one end is accompanied by the dissolution of metal at the other end to achieve actuation (Figure 8I). Subsequently, Loget et al. [115] exploited the fact that conducting substances can undergo spatially separated redox reactions in the presence of an electric field, which can generate asymmetric bubbles at both ends of the metal, to achieve controlled actuation of a microrobot. On this basis, Bouffier et al. [182] increased the speed of movement of bubble-driven microrobots using hydroquinone solutions. This is due to the fact that the redox potential of hydroquinone is lower than that of water, leading to higher speeds by expelling it at only one end of the microrobot.

Preparation of micro- and nanorobots through the use of virgin materials is a standard procedure that offers noteworthy benefits. Firstly, this method offers a broad selection of materials that can be synthesized and tailored to the properties and structures of the initial materials, enabling the creation of customized and versatile designs for micro- and nanorobots. Secondly, the use of basic materials for developing micro- and nanorobots provides good controllability, enabling high-precision control of scale and morphology to meet diverse application requirements. Furthermore, preparing micro- and nanorobots from raw materials offers high scalability and preparation efficiency, meeting the demands of large-scale preparation and mass production. However, the use of basic materials for the preparation of micro- and nanorobots has certain limitations and drawbacks. Firstly, synthesizing and processing raw materials is relatively complex, requiring a high level of chemical synthesis and processing techniques, as well as precise process control. This can limit the feasibility and difficulty of implementing micro- and nanorobot preparation. Secondly, using raw materials directly to prepare micro- and nanorobots may encounter difficulties in terms of their purity and stability. Impurities or unstable materials can impact the performance and stability of these robots. Furthermore, preparing micro- and nanorobots from virgin materials is costly, requiring significant equipment and resource investments.

## 3. Stimulus–Response Mechanisms and Applications of Micro/Nanobots

Therefore, in order to use the micro- and nanorobots prepared by the above method in various conceivable scenarios, it is necessary to understand the stimulus–response mechanism of the micro- and nanorobots and then to establish a reasonable actuation method so that they can be moved to the target position. So far, micro-mechanisms with different stimulus–response mechanisms have been developed by modifying the physical or chemical properties of the microrobots. To provide a visual overview, Table 2 lists the microrobots that achieve motion based on different stimulus–response mechanisms. In general, microrobots can move in response to various stimuli such as heat, light, pH, ultrasound, magnetic field, biological and ionic. This section describes the different stimulus–response mechanisms and their application scenarios that enable microbots to move.

### 3.1. Thermal Stimulus Response Mechanisms

Typically, thermally driven micromotors consist of at least one temperature-sensitive material. To date, there are two common types of thermally driven micromotors: polyester fiber poly(N-isopropylacrylamide) (pNIPAM) [52,59,60,61,183], and liquid crystal elastomers (LCEs) [184]. The mechanisms of motion of these two thermally driven micromotors are quite different, with polyester fibers achieving actuation through morphological changes (expansion and de-expansion) and liquid crystal elastomers moving due to heterogeneity or disorder in the particle structure.

**Figure 9:**
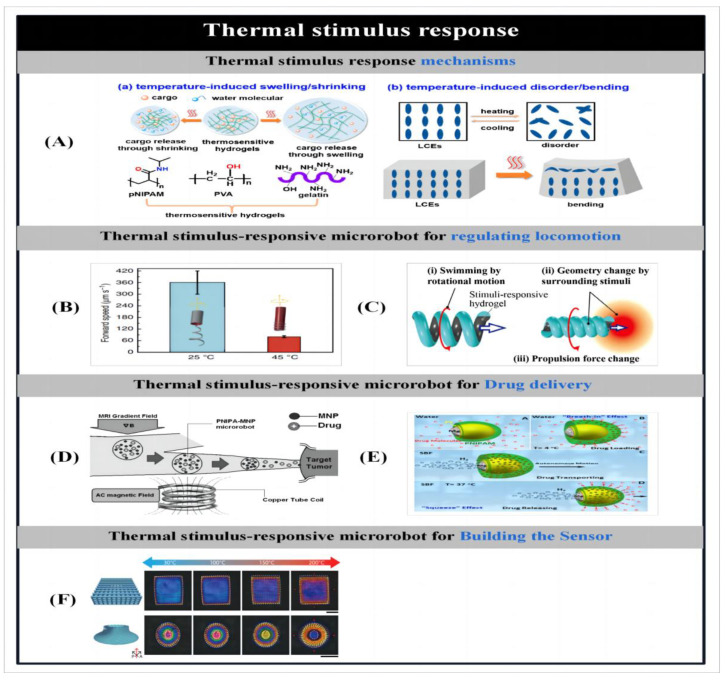
Examples of thermal stimuli-responsive microrobots. (**A**) Thermal stimulus–response mechanisms in microbots: (a) temperature-induced swelling/contraction of hydrogels; (b) temperature-induced disorder/bending of LCEs. Reproduced from Ref. [1]. Copyright 2023, the American Chemical Society. (**B**,**C**) Thermosensitive hydrogel microrobot for regulating motion. Reproduced from Refs. [183,185]. Copyright 2016, the Authors and 2020, the Authors. (**D**,**E**) Magnetically driven thermosensitive hydrogel microrobots for cancer therapy. Reproduced from Refs. [60,61]. Copyright 2011, Koninklijke Brill NV, Leiden and the Robotics Society of Japan and 2014, the American Chemical Society. (**F**) Thermally Responsive 4D Liquid Crystal Microactuator for Color Sensing. Reproduced from Ref. [184]. Copyright 2021, the Authors.

Polyester fibers, the most common type of thermosensitive hydrogel, have a much lower critical solution temperature. In an aquatic solution, changes in particle solubility due to temperature changes can lead to changes in the morphology of the polyester fibers (Figure 9A-a). If the temperature exceeds the minimum critical solution temperature, the polyester fibers will precipitate in the aquatic environment due to the breaking of hydrogen bonds. The morphology of the polyester fibers changes to a contracted state [60]. This is because polyester fibers are more sensitive to temperature changes and can be deformed by the action of thermal stimuli. This is therefore used to control the direction and speed of movement of micromotors based on thermally sensitive polyester fibers (Figure 9B) [52,183,185]. For example, Fiedler et al. [52] achieved a reversible control system for micromotors by controlling small changes in temperature. Janus particles partially coated with thermosensitive polyphosphazenes degrade the fuel in an aqueous solution containing hydrogen peroxide. As the fuel decomposes, the particles expel bubbles and move rapidly forward. The change in polymer conformation modulates the rate of bubble generation when small changes in temperature occur, resulting in greater acceleration/deceleration of the micromotor. Similarly, Yoshida et al. [185] enabled a microswimmer to sense thermal stimuli from the external environment and thus control its own motion by incorporating a thermosensitive hydrogel into a double-layered micro-spiral swimmer (Figure 9C). In addition, polyester fiber-based thermal micromotors were applied in many fields such as cancer therapy (Figure 9D) [60], drug delivery (Figure 9E) [61], tracking [59,60], in vivo imaging [59,60], and so on. As the particle structure of LCEs can be heterogeneous and disordered at different temperatures, therefore, under the effect of thermal stimulation from the surrounding environment, LCEs will undergo phase transition or produce bending (Figure 9A-b). Based on the thermal bending property of liquid crystal elastomers, del Pozo et al. [184] fabricated microactuators using liquid crystal photoresists with highly cross-linked networks. The microactuators are not only capable of controlled deformation, but also have unique polarization colors. Therefore, they can be used in the construction of sensors and micro-counterfeiting devices (Figure 9F).

### 3.2. Light Stimulus Response Mechanisms

**Figure 10:**
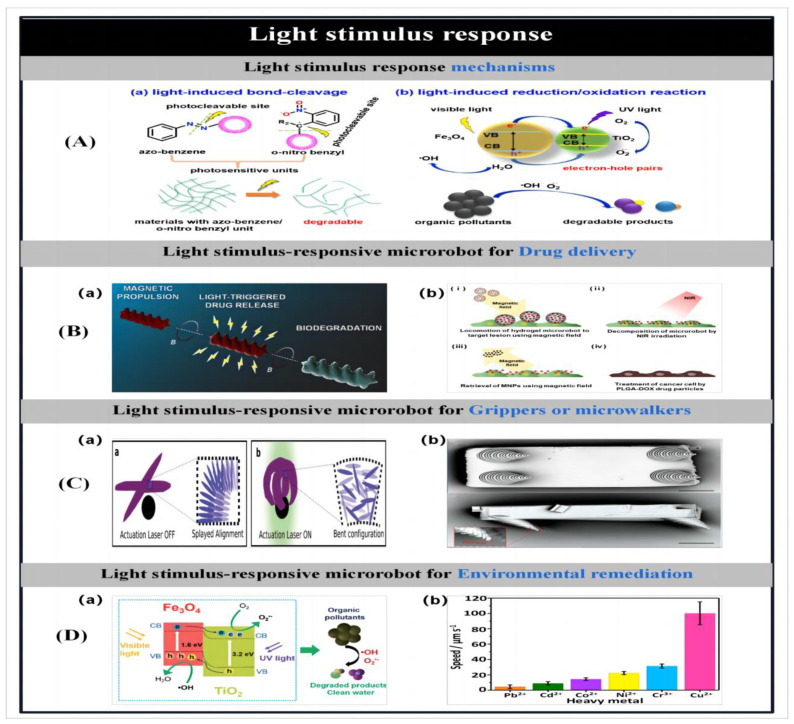
Examples of light stimuli-responsive microrobots. (**A**) Light stimulus–response mechanisms in microbots: (a) light-induced bond-cleavage; (b) light-induced oxidation/reduction reactions. Reproduced from Ref. [1]. Copyright 2023, the American Chemical Society. (**B**) Light stimulus-responsive microrobot for drug delivery: (a) UV-sensitive microrobot for drug delivery. Reproduced from Ref. [20]. Copyright 2018, the American Chemical Society; (b) hydrogel-based microrobot for near-infrared light-induced drug release. Reproduced from Ref. [27]. Copyright 2019, 2020, Elsevier B.V. (**C**) Light stimulus-responsive microrobots for grippers or microwalkers: (a) light stimulus-responsive microrobot for load capture. Reproduced from Ref. [18]. Copyright 2017, WILEY-VCH; (b) light stimulation-responsive microrobot for making a micro-walker. Reproduced from Ref. [16]. Copyright 2015, the authors. (**D**) Light stimulus-responsive microrobot for environmental remediation (a) Biohybrid microrobot composed of Fe_4_O_3_@TiO_2_ for removal of organic pollutants from water. Reproduced from Ref. [19]. Copyright 2019, the Royal Society of Chemistry; (b) metal-free micromotor composed of C_3_N_4_ for the removal of heavy metals from water. Reproduced from Ref. [28]. Copyright 2018, the American Chemical Society.

Light-driven microrobots are microrobots that are sensitive to external light stimuli. Depending on their composition and light response mechanism, light-driven microrobots can be divided into three main categories:

(i) These light-driven microrobots consist of photosensitive units such as azobenzene or o-nitrobenzene [17]. The induced action of light causes bond breaking/structural decomposition of the photosensitive units, which converts the optical momentum into the force required to move the micromotor (Figure 10A-a) [20]. For example, Bozuyuk et al. [20] prepared magnetic double-helix microswimmers with a diameter of 6 μm and a length of 20 μm via the 3D printing of a natural derivative of chitosan using two-photon 3D printing in the form of magnetic polymer nanocomplexes. The amino groups were then attached to the Adriamycin-modified swimmers by photocleavage. The microswimmers could release Adriamycin with an efficiency of 60% in 5 min under the induced effect of light. The microswimmers could be precisely controlled under the effect of an applied rotating magnetic field. The incorporation of chitosan enhances the bioadaptability and biodegradability of the microswimmers, allowing them to be rapidly degraded to non-toxic products under relevant physiological conditions (204 h). In addition, the amount of drug released in the time domain can be altered by controlling the light-induced mode, which causes the drug release to stop (Figure 10B-a).

(ii) These light-driven microrobots consist of thermo-responsive hydrogels and liquid crystal elastomers [16,18]. Under the action of photothermal heating stimuli, the structure of the thermo-responsive material is made to deform (expansion/contraction/disorder/bending), leading to the realization of movement by the microrobot (Figure 9A). Inspired by the human hand, Martella et al. [18] designed a micro-equivalent capable of capturing trace elements (Figure 10C-a). The light-sensitive micromanipulator can not only be remotely controlled under the effect of light stimulation, but can also create conditions in its own environment that automatically trap particles according to its optical properties. By incorporating liquid crystal elastomers into the micromanipulator, the light- and heat-sensitive properties of the soft material are exploited, allowing the micromanipulator to move only under the stimulation of ambient light.

(iii) These light-driven microrobots consist of a semiconductor with an appropriate energy band gap. Under the catalytic effect of light, the semiconductor undergoes an appropriate oxidation/reduction reaction, resulting in the movement of the micromotor (Figure 10A-b) [19]. Mushtaq et al. [19] fabricated a Fe_3_O_4_-TiO_2_ magnetic micro-helical robot using biotemplate and sol–gel techniques. Under UV stimulation, the particles can effectively degrade organic pollutants in water (Figure 10D-a). Under the stimulation of visible light, the particles could remove the RhB dye from the water within 75 min, and the degradation efficiency reached 97%. Meanwhile, when the microrobot was subjected to photocatalytic degradation with an applied continuous magnetic field, the organic pollutants in the water could be degraded within 40 min with 99% decontamination efficiency.

To date, light-driven micromotors have been widely used in many applications such as drug delivery (Figure 10B-b) [17,21,22,23,24,25,27], microwalkers (Figure 10C-b) [16], fabrication of flexible electronic devices [26], and environmental remediation (Figure 10D-b) [28].

### 3.3. Acoustic Stimulus Response Mechanisms

**Figure 11:**
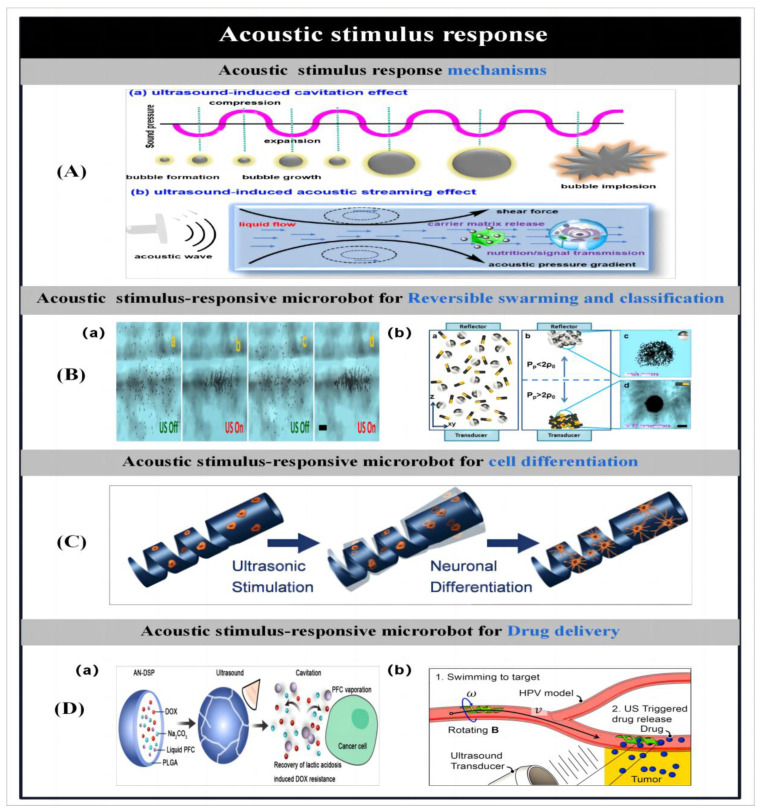
Examples of acoustic stimuli-responsive microrobots. (**A**) Acoustic stimulus response mechanisms in microbots: (a) US-induced cavitation effect; (b) US-induced streaming effect. Reproduced from Ref. [1]. Copyright 2023, the American Chemical Society. (**B**) Acoustic stimulus-responsive microrobot for reversible swarming and classification: (a) reversible clustering and dispersion of ultrasound-triggered Pt-Au nanowire motors; (b) ultrasound-triggered separation of different types of catalytic nanomotors.Scale bar, 20 μm. Reproduced from Ref. [7]. Copyright 2015, the American Chemical Society. (**C**) Ultrasound-sensitive microrobot for neuron-like cell delivery and neuronal differentiation. Reproduced from Ref. [5]. Copyright 2019, the Royal Society of Chemistry. (**D**) Acoustic stimulus-responsive microrobot for drug delivery: (a) ultrasound-sensitive microrobot for alleviating drug resistance due to lactic acidosis induced by tumor tissue. Reproduced from Ref. [2]. Copyright 2020, the Royal Society of Chemistry. (b) Acoustic–magnetic combination allows microrobots to rapidly release drugs in a short cycle time. Reproduced from Ref. [4]. Copyright 2020, Elsevier B.V.

Ultrasound serves as an important external stimulus that enables microrobots to achieve locomotion. The reason for the widespread use of microrobots in the biomedical industry can be attributed to the following two reasons: (i) Ultrasound is characterized as safe, minimally invasive and biocompatible. (ii) By modulating the ultrasound beam/force, it allows precise positioning at the micro- and nanoscale in tissues at deeper depths. As one of the important means of propelling micro- and nanorobotics, ultrasound can not only move particles by generating longitudinal forces, but also by inducing mechanical forces/local thermal effects to move particles [2]. Under the stimulation of ultrasound, the driving mechanism of microrobots can be mainly classified into two types: (i) Cavitation effect (Figure 11A-a). The cavitation effect refers to the formation, oscillation, growth, contraction and collapse of bubbles (cavities) under the action of ultrasonic field. Tiny bubbles stimulated by alternating periodically fluctuating ultrasonic waves generate high temperatures and pressure at the moment of bubble collapse, and the local high-energy part triggers physicochemical changes. For example, Lu et al. [6] proposed a method for the rapid growth and movement of dandelion-like microclusters composed of catalytic tubular microengines. The self-generated bubbles of the microtubes oscillate in the presence of ultrasound waves, allowing the microcluster to overcome the resistance created by a large and disorganized number of bubbles within a single cell. Driven by ultrasound, individual manganese dioxide tubular micromolecules float rapidly in a surfactant-free hydrogen peroxide solution, led by self-generated oxygen bubbles. A large bubble nucleus formed by the fusion of multiple microbubbles was excited and oscillated by the acoustic wave, and the resulting locally enhanced acoustic field attracted more individual micromotors to gather around it, forming a dandelion-like microcluster. Subsequently, Xu et al. [7] used this property to complete the classification of different types of nanomotors (Figure 11B-b), based on the fact that different types of micromotors will cluster in different regions (Figure 11B-a). (ii) Acoustic flow effect (Figure 11A-b) [3]. The acoustic flow effect is based on the absorption of acoustic waves by the fluid, which generates a driving force and at the same time promotes the generation of an acoustic pressure gradient, thus accelerating the heat and mass transfer process.

So far, acoustically driven microrobots have been widely used in many scenarios such as targeted drug delivery [2,3,7], delivery and differentiation of neuron-like cells (Figure 11C) [5], and cluster control [6,7]. For example, to reduce drug resistance due to lactic acidosis induced by tumor tissue, Meng et al. [2] developed an acoustic-driven alkaline microrobot (AN-DSP) consisting of PLGA nanoparticles containing Adriamycin (DOX), NaCO_3_ and perfluorocarbons (PFCs) to reverse lactic acidosis-induced drug resistance. The AN-DSP is able to rapidly respond to external ultrasound stimuli and rapidly release sodium carbonate to neutralize lactic acidosis, thereby enhancing DOX sensitivity in vitro and in vivo. In response to external ultrasound stimulation, AN-DSP can autonomously accumulate in tumor tissue through enhanced permeability and retention effects. In doing so, it specifically disrupts the acidic environment of the tumor tissue, ultimately limiting tumor growth with minimal side effects (Figure 11D-a). Subsequently, to further reduce the stimulus response time and accelerate drug release, Darmawan et al. [4] used helical micromotors prepared using a monolayer self-folding technique as a controllable drug release system, so that they could be manipulated by an applied magnetic field for efficient drug release. The helical micromotor was able to rapidly change shape under proton/non-proton stimulation and follow a predetermined path to the target location under the action of a rotating magnetic field generated by an electromagnetic drive system. Subsequently, under acoustic stimulation, the non-covalently bound anticancer drug is released in a very short time cycle (Figure 11D-b).

### 3.4. pH Stimulus Response Mechanisms

**Figure 12:**
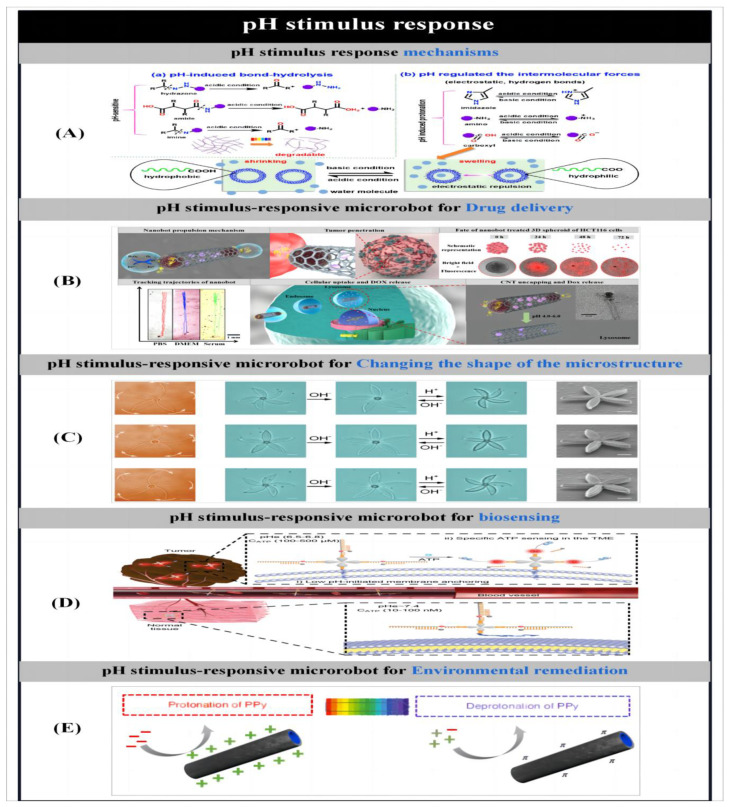
Examples of pH stimuli-responsive microrobots. (**A**) pH stimulus–response mechanisms in microbots: (a) pH-induced bond-hydrolysis; (b) pH-regulated intermolecular forces. Reproduced from Ref. [1]. Copyright 2023, the American Chemical Society. (**B**) Oxygen bubbles propel nanorobots to deeply penetrate tumor tissue and release anticancer drugs in the presence of pH. Reproduced from Ref. [187]. Copyright 2020, the authors. (**C**) pH change induces structural changes in microbots. Reproduced from Ref. [186]. Copyright 2019, Wiley-VCH. (**D**) ATP-sensing microbots in acidic tumors. Reproduced from Ref. [67]. Copyright 2019, Wiley-VCH. (**E**) Removal of estrogenic contaminants from water using pH-sensitive microrobots. Reproduced from Ref. [62]. Copyright 2020, the authors.

The driving mechanisms of pH stimulus-responsive micromotors can be divided into two main categories based on the pH-sensitive materials:

(i) The pH-induced hydrolysis of covalent bonds such as imine bonds, ester bonds, ligand bonds, acetal/ketone bonds, etc. (Figure 12A-a), at the pH-sensitive sites of the microbots induces the decomposition of pH-sensitive complexes in the microbots to achieve movement. For example, Andhari et al. [187] designed a multicomponent magnetic micro- and nanorobot for targeted cancer therapy. It was made by chemically binding magnetic Fe_3_O_4_ nanoparticles, anti-epithelial cell adhesion molecule antibodies (anti-EpCAM mAb), to multi-walled carbon nanotubes loaded with the anticancer drug adriamycin hydrochloride (DOX). The catalytic property allows the micro- and nanorobots to be self-propelled in complex biofluids. The magnetic property allows the micro-nanorobot to achieve magnetic navigation in the presence of an external magnetic field, facilitating the release of DOX into the lysosomal intercompartment within human colorectal cancer (HCT16) cells through the Fe_3_O_4_ particle inlet (Figure 12B).

(ii) The pH induces the expansion or contraction of the microbots by modulating intermolecular forces such as electrostatic or hydrogen bonding within the pH-sensitive units in the microbots (Figure 12A-b), resulting in changes in their physical structures. For example, Hu et al. [186] achieved microbionic 4D printing of pH-responsive hydrogels in the spatio-temporal domain using DLW. The size of the printed structures was in the microscale range (<100 μm) and the response time to external stimuli could be reduced to the millisecond range (<500 ms). By exploiting the ability of the hydrogel to expand, contract and twist in response to pH stimuli, the printed microstructures are able to undergo multi-degree-of-freedom shape transformations (Figure 12C). In the same vein, the use of reconfigurable micromachines to enable structural deformation of microrobots is also a common tool. Reconfigurable micromechanisms are highly sensitive to environmental changes, and nanofabrication methods with intricately designed micromechanisms can improve the controllability of their shape changes, such as bending, folding and twisting, while minimizing these response times. However, due to the lack of available materials and effective preparation techniques, it is very difficult to prepare 3D structures with a high degree of freedom for shape change at the microscale. Jin et al. [170] introduced a 4D microprinting method that realizes 3D–3D shape-changing micromechanisms in a single material and a single step. Using DLW, complex stimulus-responsive hydrogels are dispensed into arbitrary 3D structures with sub-millimeter features, and the exposure dose of a programmed millisecond laser beam is used to adjust the cross-linking density, stiffness and degree of expansion/shrinkage of the material to achieve complex 3D structural transformations.

pH stimulus-responsive micro motors can be widely used not only for targeted cancer therapy [58,63,64,65,66], biosensing (Figure 12D) [67], and changing the shape of microrobots [173,174], but also for environmental improvement. For example, Dekanovsky et al. [62] developed programmable microrobots with polypyrrole (PPy) as the outer functional layer and Pt as the inner catalytic layer, to which ferric tetraoxide paramagnetic particles were added. The catalytic properties of the Pt layer enabled the microrobots to achieve self-drive, and the paramagnetic particles enabled the microrobots to respond to an applied magnetic field to achieve The catalytic properties of the Pt layer allowed the microrobot to be self-actuated, while the paramagnetic particles allowed the microrobot to move in a directional manner in response to an applied magnetic field, thus allowing the microrobot to efficiently remove estrogenic contaminants. As the pH of the tested water changes, the charge on the surface of the microbots gradually changes, leading to affinity modulation. As the microbots move through the solution, they clump together estrogen fibers. The estrogenic fibers are then compiled into a macroscopic web on the functionalized outer surface, resulting in the removal of estrogenic contaminants from the water (Figure 12E).

### 3.5. Magnetic Stimulation Response Mechanisms

**Figure 13:**
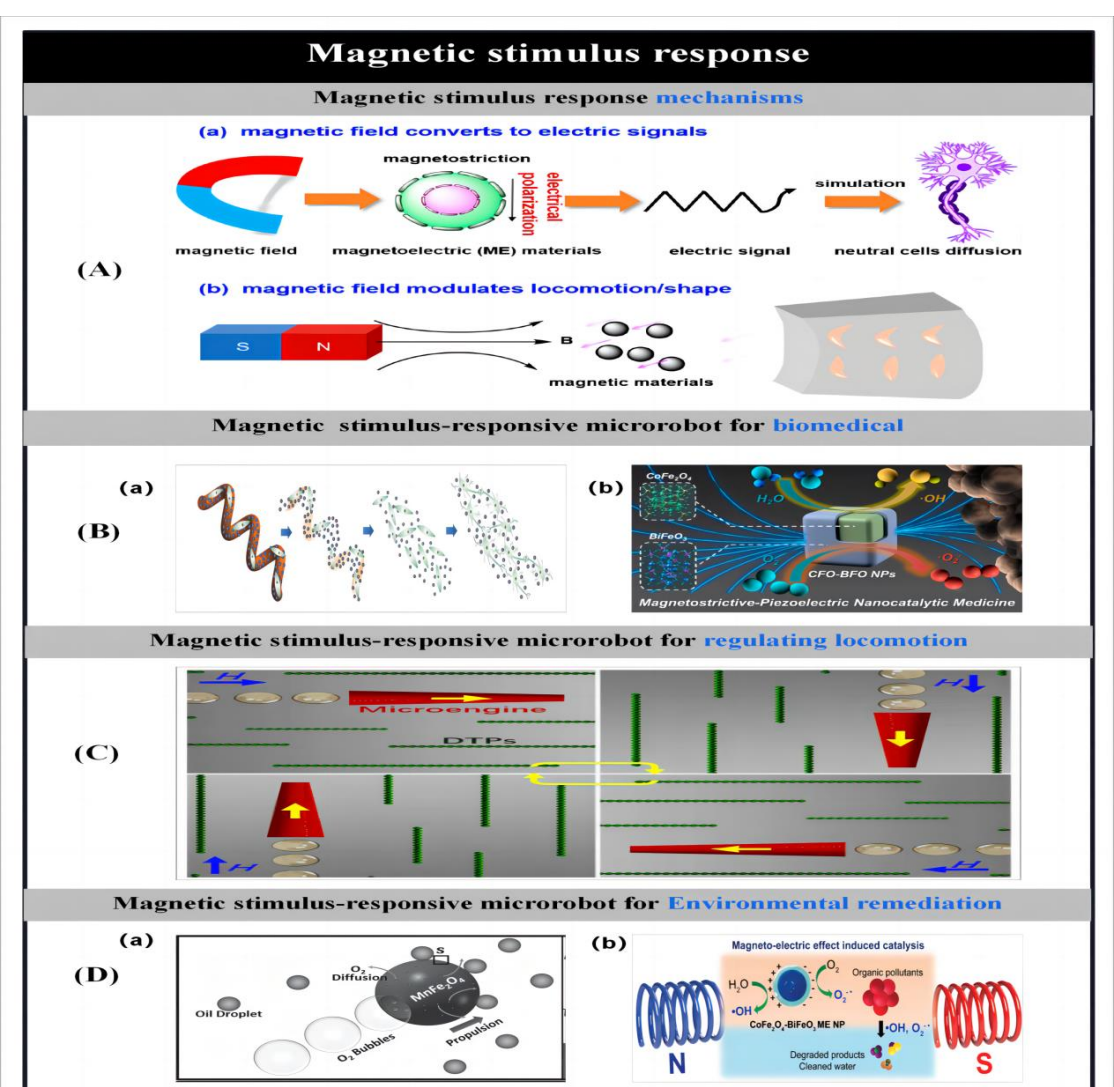
Examples of magnetic stimuli-responsive microrobots. (**A**) Magnetic stimulus response mechanisms in microbots: (a) magnetic field modulating the locomotion/shape; (b) magnetic field transferred into electric signals. Reproduced from Ref. [1]. Copyright 2023, the American Chemical Society. (**B**) Magnetic stimulus-responsive microrobot for biomedical: (a) magnetic stimulation-responsive microrobots for cell differentiation. Reproduced from Ref. [9]. Copyright 2020, Wiley-VCH; (b) magnetic stimulation-responsive microrobots for tumor therapy. Reproduced from Ref. [14]. Copyright 2021, the American Chemical Society. (**C**) Using magnetic fields to tune the motion behavior of a microrobot. Reproduced from Ref. [11]. Copyright 2018, the American Chemical Society. (**D**) Magnetic stimulus-responsive microrobot for environmental remediation: (a) removes oil droplets from water with a jug-shaped micromotor. Reproduced from Ref. [12]. Copyright 2015, Wiley-VCH; (b) microrobot with core-shell structure degrades organic pollutants in water in the presence of a magnetic field. Reproduced from Ref. [13]. Copyright 2019, Wiley-VCH.

Magnetic fields have been widely used to drive microrobots due to their biocompatibility, remote wireless control, ease of integration and high accuracy. Mechanisms for driving microscale matter using magnetic fields can be classified into the following three main types:

(i) Magnetic materials that are added to the existing non-magnetic microstructures. Such composite microstructures possessed magneto-electric properties, allowing directional control based on the original free motion (Figure 13A-a) [9,11]. For example, Dong et al. [9] infused multiferroic nanoparticle complexes (MENPs) into the helical soft structure of light-curable gelatin-methacrylic alcohol (GeIMA)-based hydrogel microswimmers prepared via DLW, which equipped the microswimmers with magnetoelectric properties. Under the effect of magnetic stimulation, it enabled the MENPs to deliver bioactive chassis to the target location, thereby inducing neuron-like cell differentiation (Figure 13B-a). In addition, the nanoparticle complexes could also convert the magnetic field stimulation into an electrical signal (Figure 13A-b), which was then used as a magnetically mediated electrical trigger for neuron-like cells. Subsequently, Yang et al. [11] achieved reversible motion control of microstructures in ferromagnetic fluids by using dynamic terrain paths formed by superparamagnetic nanoparticles in response to external magnetic stimuli. The dynamic terrain paths change the motion direction of the micromotor by applying anisotropic resistance on the autonomously moving micromotor. Due to the different directions and lengths of the dynamic terrain paths, rapid reversible assembly can be accomplished under the action of an external magnetic field. Thus, the motion direction and trajectory of the micromotor are controlled, which enables the micromotor to realize a variety of different modes of motion trajectories, such as circular, elliptical, semi-sinusoidal and sinusoidal. This motion control strategy no longer eliminates the dependence on external stimuli by adjusting the mode of action and relevant parameters of external stimuli (Figure 13C).

(ii) A magnetic field is directly used to drive microrobots composed entirely of magnetic materials (iron, nickel and other magnetic materials) [13,14]. For example, Mou et al. [12] demonstrated the removal of organic pollutants from water by a micromotor composed of pot-shaped hollow manganese ferrite particles. In an oil droplet consisting of trichloromethane and hexane, nanoparticles consisting of hydrophobic manganese ferrite with oleic acid are assembled into a densely packed layer of particle shells due to hydrophobic interactions between the particle surfaces. As the encapsulated oil droplets are continuously vaporized to form high pressure bubbles, the compact shell layer ruptures. A single hole is formed in the shell, creating an asymmetric pot-shaped micro engine. Oxygen molecules produced by the micromotor through catalytic decomposition of hydrogen peroxide preferentially nucleate on the concave surface of the inner layer and grow into bubbles that are continuously ejected through the holes to drive the micromotor, effectively removing the oil droplets (Figure 13D-a).

(iii) By combining magnetic actuation with other actuation methods, the direction of motion of the particles can be changed. For example, Ren et al. [8] introduced bubble-based acoustic-field-driven microswimmers that can move autonomously in three dimensions, and that selectively transport individual synthetic glia and mammalian cells in relatively dense populations without the need for labelling, surface modification or impact on the surrounding material. In a MHz acoustic field, the microswimmer is subjected to two forces: a secondary Buell–König force and a locally generated acoustic current drive, the combination of which allows the microswimmer to move freely in free space under the influence of the magnetic field.

Up to now, since magnetic fields are transparent and harmless to biological tissues, they have been widely used to drive microrobots in biomedical applications. Kato et al. were the first to propose the use of micro- and nanorobots for magnetic guidance of selective chemotherapy for cancer [188]. From albumin magnetic microbeads to ferromagnetic mitomycin microcapsules, these drug delivery systems were localized to tumor sites in vivo in the presence of a magnetic field, demonstrating that overcoming the side-effects of systemic chemotherapy is a key factor in magnetically activated drug delivery systems. Therefore, it is common to incorporate magnetic layers or additional magnetic nanoparticles into the microstructures, allowing the microbots to respond to external magnetic fields. For example, Nelson et al. enabled real-time tracking and control of microbots by fluorescence visualization while swimming in the abdominal cavity of mice through incorporating near-infrared fluorescent moieties into magnetically driven artificial microswimmers [189]. The spiral-structured microswimmers achieve movement under the action of a magnetic field generated by an alternating current. The motion behavior of the microswimmers can be controlled by adjusting the size and direction of the magnetic field. Subsequently, in order to obtain the optimal manipulation of magnetic nanoparticles in the presence of a magnetic field, Martel et al. demonstrated the motion of PLGA-based particles loaded with the anticancer drug DOX and iron cobalt nanoparticles [190]. The magnetic nanoparticles enabled the particles to be delivered to the tumor targeting location in response to an external magnetic field and the systemic concentration of Adriamycin was minimized due to local drug release. This composite particle was able to maneuver 4 cm below the skin in response to an external magnetic field, delivering the drug precisely to the targeted location and releasing it in a controlled manner. To further confirm the phenomenon of drug localization in the human body, Price et al. used magnetic particles to encapsulate DOX and deliver it to the lungs for lung cancer treatment [191]. In the presence of a magnetic field, particles carrying anticancer drugs could achieve specific localization in vivo.

To date, the use of magnetic fields to drive microrobots has not only been applied to induced cell differentiation, tumor therapy (Figure 13B-b) [14], cell manipulation [15], locomotion control and environmental remediation (Figure 13D-b) [13], but can also be used to change the shape of microrobots. As a deformable material, origami is characterized by its polymerizability, versatility and tunability. To further expand the applications of origami, Novelino et al. [10] combined the geometric and mechanical properties of bistable Kresling modes with magnetically responsive materials to produce magnetically controlled origami systems. In the presence of an applied magnetic field, the miniature origami robot is able to achieve unconstrained actuation at controlled speeds and can instantly latch onto a shape, enabling shape transformation.

### 3.6. Biological Stimulus Response Mechanisms

With the increasing maturity of nanotechnology, the combination of composite particles with biological cells to construct biohybrid microrobots has become mainstream. Typically, biohybrid microrobots are composed of biodegradable hydrogels (e.g., GelMA) [8,30,31], biological cells (e.g., neutrophils [32]), microbial tissues (e.g., bacteria and algae [33]), and biomolecules (DNA molecules [34]). These components are biodegradable and bioadaptable, which not only enables micro- and nanorobots to cross the blood–brain barrier in the human body, but also avoids immune tracking, thus enhancing the application of microrobots in vivo [34]. For example, Zhang et al. [32] reported a neutrophil-based microrobot that can actively deliver drug loads to malignant glioma sites in the human body. The neutrophils constructed the neutral robot by phagocytosing *E. coli*-coated drug-loaded magnetic nanogels, in which the wrapping of the *E. coli* film not only enhances phagocytosis efficiency but also prevents drug leakage within the neutrophils. Under the influence of a rotating magnetic field, the neutrophils exhibit controlled locomotion within the vasculature and actively aggregate in the brain. They then cross the blood-brain barrier in the positive chemotactic direction of the inflammatory factor gradient and reach the area where the tumor cells are located to release the drug, thus inhibiting the value of the tumor cells.

**Figure 14:**
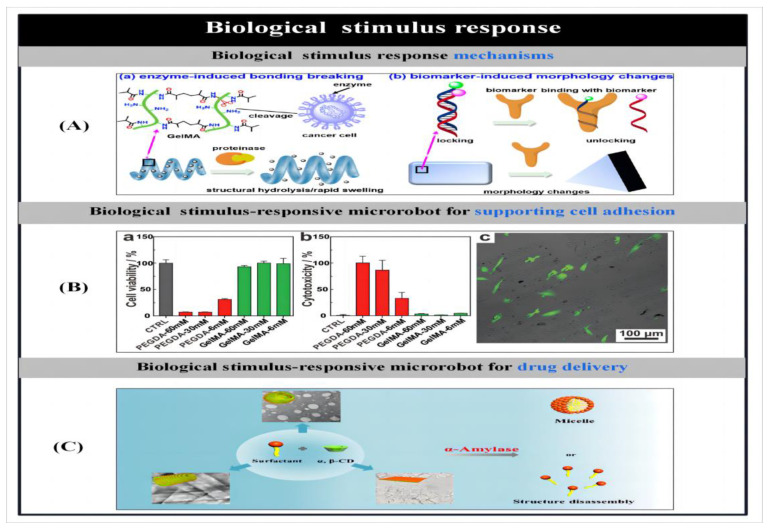
Examples of biological stimuli-responsive microrobots. (**A**) Biological stimulus–response mechanisms in microbots: (a) enzyme-induced bond breaking; (b) biomarker-induced morphology changes. Reproduced from Ref. [1]. Copyright 2023, the American Chemical Society. (**B**) GeIMA-based microrobots for supporting cell adhesion and growth. (a) Cell viability after 24 h of incubation with GelMA and PEGDA solutions using the MTT assay: cells cultivated on tissue culture plate were set as control (CTRL); each data set corresponds to mean ± SD (n = 4). (b) Cytotoxicity of GelMA and PEGDA solutions after 24 h of incubation using the LDH assay; each data set corresponds to mean ± SD (n = 4). (c) A confocal microscopy image of live-dead stained human fibroblasts seeded on GelMA helices after 12 h. The live and dead cells are green and red, respectively. Cells were highly viable and preferably attached to GelMA structures.Reproduced from Ref. [31]. Copyright 2018, Wiley-VCH. (**C**) Enzyme-responsive molecules for drug delivery. Reproduced from Ref. [35]. Copyright 2021, the American Chemical Society.

The movement mechanisms of biostimulus-responsive micro- and nanorobots can be classified into two categories: (i) enzyme-induced bond breaking (Figure 14A-a). For example, Wang et al. [31] prepared biodegradable soft spiral microswimmers by DLW using non-toxic photo-crosslinked hydrogel methacryloyl (GeIMA). The surface of the micro swimmers was made magnetically responsive by modifying them with magnetic nanoparticles. The flexible micro-spiral swimmers were able to spiral forward at a higher forward speed above the step-out frequency compared to conventional rigid spiral robots. In addition, GeIMA have lower cytotoxicity and can be completely degraded by collagen compared to polyethylene glycol acrylate-based microbots. Furthermore, they can support cell attachment and growth and are gradually digested by enzymes released by the cells during culture (Figure 14B). (ii) The morphology of chemically/physically modified biomolecules/biological organisms/biological cells is altered (Figure 14A-b). Liu et al. [34] described a stable DNA nanorobot (TDA). It is capable of achieving controlled conformational changes in response to the stimulation of epithelial cell adhesion molecules specifically expressed in the tumor cell cycle, and TDA has lower cytotoxicity and target specificity. Thus, TDA is not only effective against nuclease catalysis, but also has the ability to precisely target EpCAM-positive cells. These microrobots have been widely used in drug delivery (Figure 14C) [8,30,35], cell adhesion [31] and early cancer diagnosis [33,34].

### 3.7. Ionic Stimulus Response Mechanisms

**Figure 15:**
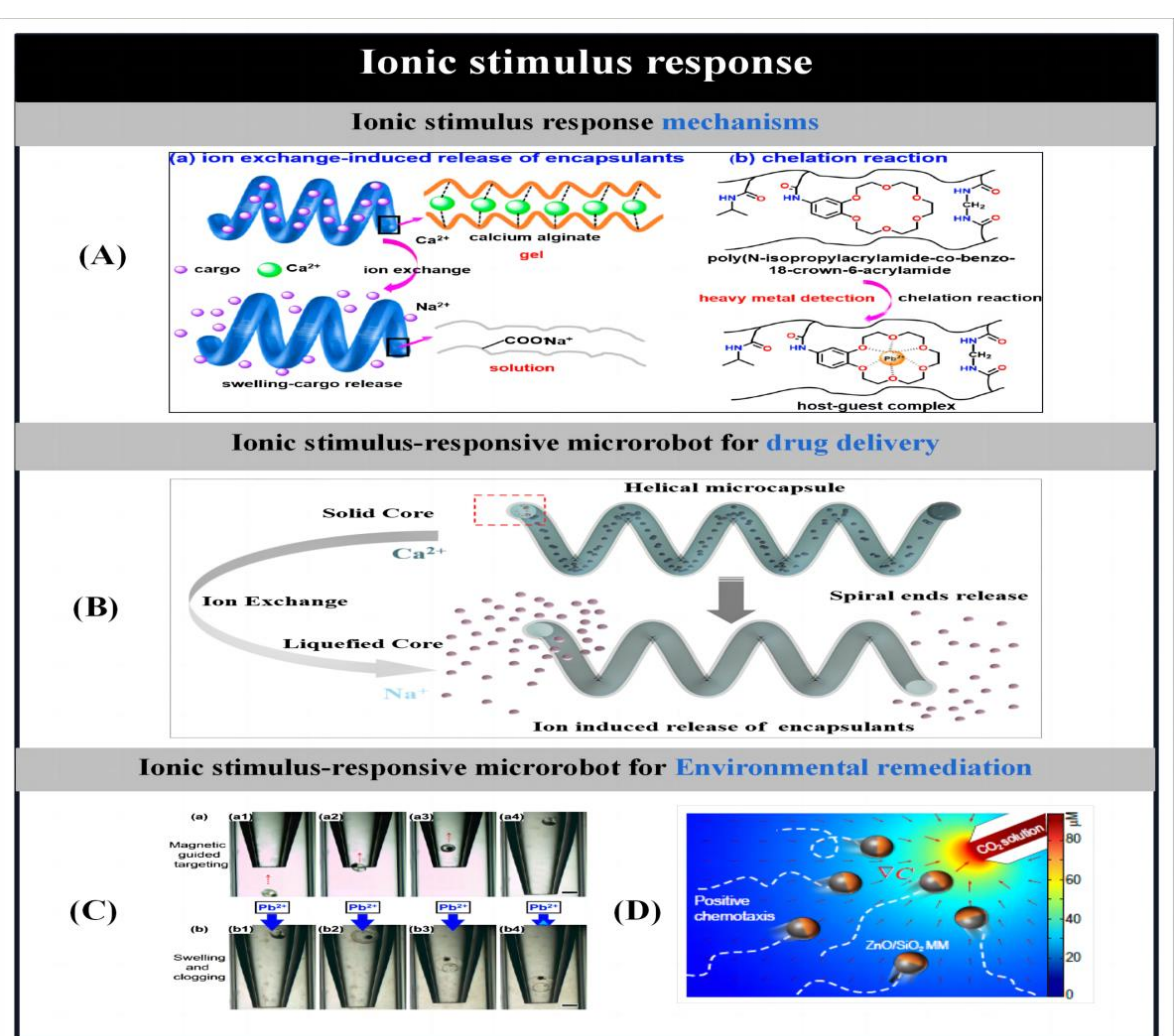
Examples of ionic stimuli-responsive microrobots. (**A**) Ionic stimulus–response mechanisms in microbots: (a) ion-induced phase changes; (b) ion-induced chelation. Reproduced from Ref. [1]. Copyright 2023, the American Chemical Society. (**B**) Ion exchange microrobots for drug delivery. Reproduced from Ref. [57]. Copyright 2021, the Royal Society of Chemistry. (**C**) Ion-triggered microbots for monitoring lead ions in water. (a) Magnetic-guided targeting process of the microactuator into the microtube. (b) Clogging of the microtube by Pb^2+^-responsive swelling of the microactuator in response to flow of Pb^2+^-contaminated solution. Scale bars are 200 μm. Reproduced from Ref. [68]. Copyright 2013, the American Chemical Society. (**D**) Zinc oxide-based micromotor for detecting carbon dioxide in water. Reproduced from Ref. [69]. Copyright 2021, the authors.

In addition to the use of external stimulus effects, changes in the concentration of some of the major ions in the solution (calcium/sodium ions [57] and lead ions [68]) can also affect the motor behavior of the microbots. Ion concentration changes that affect microbot memory can be classified into two types: (i) Ion changes that induce phase changes in the gel or solution in which the microbots are located (Figure 15A-a) [57]. (ii) Based on ion chelation, ions can induce morphological changes such as expansion/contraction [68] in microbots (Figure 15A-b). The ion equilibrium state and dynamic ion-responsive behavior can be regulated by adjusting the ion power and ionization during the microbot’s motion, which in turn controls the microbot’s motion behavior. Ion-responsive microbots have been widely used in drug delivery [57], environmental remediation [68,69] and other fields.

Due to the high stability, good bioadaptability and strong mechanical properties of alginate and chitosan, Liu et al. [57] compounded alginate/chitosan/alginate with Fe_3_O_4_ nanoparticles to form a multilayered structure of helical microrobots for drug loading and release. As the ion exchange of calcium and sodium ions in the hydrogel network induces a liquefaction effect, the sol-solution undergoes a phase transition, resulting in the swelling of the microcapsules (Figure 15B). Thus, under the action of ionic stimulation and an external magnetic field, the microrobot delivers the drug to the target site for release.

In addition, ion-stimulated responsive microengines have important environmental applications such as heavy metal detection [68] and carbon dioxide detection [69]. Some heavy metal ions not only cause serious environmental pollution, but also exhibit high toxicity to the human body after bioaccumulation. Lead ions have become one of the most common heavy metal pollutants in daily life due to their non-biodegradability and harmful accumulation in the environment. In order to achieve the detection of lead ions in aquatic solutions, Liu et al. [68] prepared a lead ion-sensitive microrobot. The volume of the pNIPAM hydrogel was rapidly expanded due to the formation of a host–guest complex between the hollow crown ether and lead ions. This effectively prevented the contaminated lead ion solution from leaking out of the microfluidic channel (Figure 15C). In addition, Mou et al. [69] fabricated a ZnO-based micromotor. Due to the self-diffusion electrophoretic motion of ZnO in the electrolyte in the presence of H^+^ provided by CO_2_ (Figure 15D). Therefore, its high sensitivity to CO_2_ in water can be used to detect CO_2_ in water.

### 3.8. Multi-Stimulus Response Mechanism

Unrestrained microrobots have many applications in different fields. However, their small size makes it difficult to manipulate them accurately in their environment. In addition, they can only respond to a single stimulus and are unable to achieve controlled structural deformation in response to different stimuli. To respond to a variety of physical or chemical stimuli in complex physiological environments, multi-stimulus responsive microrobots are used. Often, a variety of responsive materials with different functions are integrated into a single particle to achieve a variety of different functions. The driving mechanism of multi-stimulus responsive microrobots is to incorporate various multifunctional components into the microrobot [179,192] so that it can respond to multiple external stimuli, rather than just a single stimulus, and generate the appropriate deformation/phase change behaviors to achieve locomotion [193].

**Figure 16:**
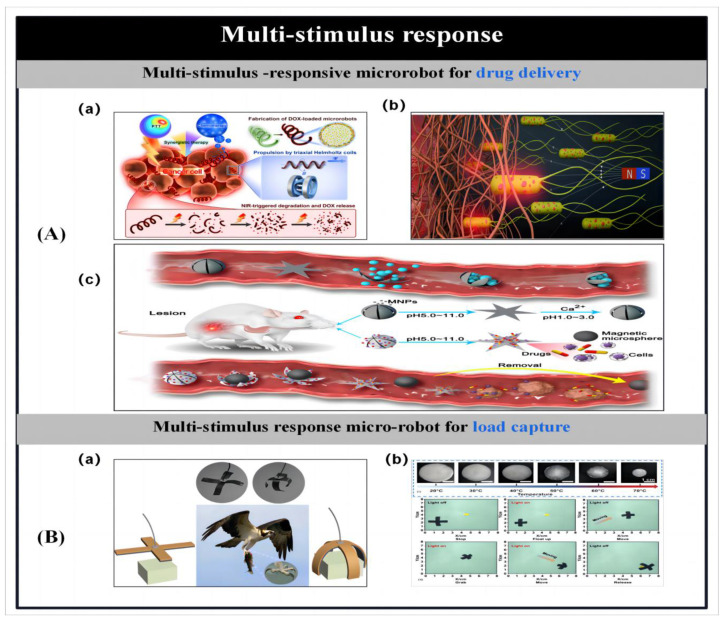
Examples of multi-stimulus–response microrobots. (**A**) Multi-stimulus-responsive microrobot for drug delivery: (a) (Pd@Au)/Fe_3_O_4_@Spirulina microrobots release drugs triggered by near-infrared light/acidic pH. Reproduced from Ref. [179]. Copyright 2019, the American Chemical Society; (b) bacterial biohybrid microrobots deliver drugs triggered by magnetic field/near-infrared light. Reproduced from Ref. [180]. Copyright 2022, the authors; (c) magnetically driven, pH/Ca^2+^ responsive microrobot for drug delivery. Reproduced from Ref. [195]. Copyright 2021, the authors. (**B**) Multi-stimulus response microrobot for load capture: (a) intelligent microbots capture loads in response to multiple stimuli such as humidity, temperature and light. Reproduced from Ref. [189]. Copyright 2019, the authors; (b) NIPAAm/AAm/PEGDA/Carbon nanoparticle hybrid microrobots capture loads in response to light and temperature stimulation. (i) The change in size during the swelling process of the hydrogel, the scale bar is 1 cm. (ii) The whole grabbing and moving process of a printed gripper submerged in water. Reproduced from Ref. [197]. Copyright 2019, the authors.

For example, Wang et al. [179] first synthesized core-shell Pd@Au nanoparticles with photothermal conversion function via electrodeposition inside spirulina cells. Subsequently, iron tetraoxide nanoparticles were deposited on the surface of the obtained (Pd@Au)@Sp particles by a sol–gel process, which enabled the microbots to have a magnetic actuation function. Finally, the anti-cancer drug Adriamycin was loaded onto the surface of the composite microrobot to make it chemotherapeutic. This biohybrid microrobot not only has an efficient propulsion performance with a maximum speed of 526.2 μm/s under a rotating magnetic field, but also has an enhanced synergistic photochemical therapeutic effect. In addition, it can be structurally decomposed into individual particles under near-infrared light irradiation for pH and near-infrared light-induced drug release (Figure 16A-a). Similarly, Akolpoglu et al. [180] incorporated magnetic nanoparticles and nanoliposomes loaded with photothermite and chemotherapeutic molecules into *E. coli* with 90% efficiency to form a bacterial biohybrid. This biohybrid microrobot not only retained its original motility, but was also able to navigate through the biological matrix and colonize tumor spheroids in the presence of an applied magnetic field. Subsequently, drugs were released on demand under near-infrared light stimulation (Figure 16A-b). However, when targeting the tumor region, the hypoxic conditions in the region where the tumor is located can make the targeted delivery of the drug extremely inefficient, making the treatment less effective. For this reason, Sridhar et al. [194] proposed a two-dimensional poly(heptazine imide) (PHI) CN particle as a light-driven microswimmer that can move at high speeds in multi-component ionic solutions with concentrations up to 5 M and without dedicated fuel. The particles have a high ion tolerance due to the good interaction between the particle configuration and structural nano-voids and the nature of the light ions. This helps in the interaction of ions in high salt solutions. The nanopores on the microswimmers have been used to load the anti-cancer drug Adriamycin with up to 185% loading efficiency and can be light-triggered for controlled on-demand release under different pH conditions. By exploiting the inherent, environmentally sensitive and light-induced charge storage properties of PHI, the microbots were able to achieve light-triggered, enhanced Adriamycin drug release under hypoxic conditions, enabling targeted therapy in hypoxic regions of tumors.

Currently, multi-stimulus responsive microrobots have been widely used in biomedical applications such as drug delivery (Figure 16A-c) [179,180,192,193,194,195]. In addition, multi-stimulus responsive microrobots are made of composite materials with different functions. Their shape can be transformed under the action of different external stimuli, and thus can be used to make grippers for capturing substances. For example, Dong et al. [196] fabricated smart microactuators with precise patterns on graphene oxide films by a hydrogel microstamping technique, which not only have the ability to change shape programmably, but also respond to a variety of stimuli such as humidity, temperature and light. Such programmable micro-actuators can mimic the claws of a hawk grasping a plate or crawling like an inchworm, and they can wrap around and grasp the axes of a flower according to its geometry (Figure 16B-a). By adjusting the intensity and direction of the NIR light, the hydrogel absorbs/loses water. This results in the rapid deformation of the hydrogel structure in a relatively short time, giving it the ability to grip loads (Figure 16B-b) [197].

## 4. Conclusions and Outlook

This paper presents a thorough examination of the current progress in the preparation methods and stimulus–response mechanisms for micro- and nanorobots, as well as their applications in biomedicine and environmental remediation. The aim of this review is to provide objective and concise information in a logically structured and clear manner, whilst adhering to the conventional format and language expected of academic writing. It includes an analysis of the fundamental design of microrobots, covering their material compositions and driving mechanisms subject to various stimuli. Additionally, given the versatile nature of microscale entities in diverse fields, this paper categorizes and analyzes the preparation procedures and operative mechanisms used for microrobots with varying materials and structures.

They can be configured with diverse and flexible shapes, controlled motion, and robust mechanical power. Microrobots have exhibited the potential to replicate biological tissue, conduct targeted drug delivery, and diagnose cancer. Nevertheless, numerous obstacles impede their practical application. Microrobots possess the ability to carry out specific assignments in intricate, mobile environments. This dissertation will outline the present challenges encountered by microrobots and offer likely resolutions (Table 3).

(i) Some functionalized microrobots have been designed to undergo complex synthesis steps, which are not suitable for scaling up and commercializing. To overcome this difficulty, microbots can be prepared utilizing 3D laser direct writing technology, which has emerged in recent years, to simplify the synthesis process. (ii) Some microbots exhibit poor sensitivity levels. Whilst microbots with bespoke stimulus–response mechanisms created through customized preparation methods may react to disease-related characteristics in organisms, these traits are not solely present in diseased regions within organisms. Nevertheless, the microbots’ limited sensitivity may compromise their selectivity in complex environments. To tackle this challenge, we can analyze the microrobot’s stimulus–response mechanism in the lesion area and optimize its structural design to enhance sensitivity, while using a reasonable actuation method. (iii) The microrobot’s mechanical properties are subpar. Although we can use adaptable materials to fabricate microrobots, we need to augment their mechanical properties to suit real-life usage. Increasing the cross-linking density while retaining its formable structure can solve this issue. Materials and chemical fuels used to construct and operate the microrobots lack biocompatibility, which restricts their application in living organisms. The usage of bioadaptable materials, including hydrogels, peptides and bio-organisms, to develop biologically safe microrobots holds the key to addressing this problem. In clinical applications, the carrying capacity of a single microrobot is restricted due to its petite size. To enhance the effectiveness of targeted drug delivery, we explored the interactions between individual microbots. Subsequently, we employed the principles of velocity matching, cohesion, and coherence to facilitate the microbots’ coordinated movement. Limiting microbots to a fixed group movement pattern leads to the improved execution of complicated tasks. (iv) In the context of using microbots for targeted drug delivery, in vivo imaging plays a crucial role in ensuring accurate targeting. To enhance targeting efficiency, implementing a feedback system to monitor and control the microbots’ movements is an essential measure to address this issue. (v) Microrobots are typically comprised of diverse materials with intricate compositions that possess biocompatibility and biodegradability in equal measure. To tackle this obstacle, biodegradable materials should be preferred in the preparation of microbots. Furthermore, for non-biodegradable materials, appropriate recycling methods should be implemented to guarantee their safe application.

## Figures and Tables

**Table 1:** Summary of fabrication techniques of micro/nanomotors.

Preparation Method	Classes	Types	Propulsion Mechanism
electrodeposition	chemical	nanowires	self-electrophoresis [77,78]
	chemical	nanotubes	bubble recoil [71,79]
	magnetic	helical	magnetic forces/torques [80,81]
	magnetic	nanowires	magnetic forces/torques [82,83,84]
	ultrasound	nanowires	acoustic pressure difference [85]
	electric	nanowires	Dielectrophoretic force [86,87]
	electric	Micro-objects	dynamic bipolar self-regeneration [88]
	chemical	Janus	bubble recoil [89]
physical vapor deposition	chemical	Janus	self-diffusiophoresis, self-electrophoresis, Bubble Propulsion, Surface tension gradients [38,46,50,90]
	magnetic	Janus	magnetically induced thermophoresis [91]
	light	Janus	diffusiophoresis induced by light [92,93]
	magnetic	helical	magnetic forces/torques [94,95]
Rolled-up Technology	chemical	nanotubes	bubble recoil [73]
	ultrasound	micro bullets	acoustic droplet vaporization [96]
	magnetic	helical	magnetic forces/torques [97]
	electric	helical	electro-osmotic flow [98]
3D direct laser writing	magnetic	helical	magnetic forces/torques [99,100]
	light	anisotropic	optical momentum conversion (OMC) [101]
Assembly of Materials	chemical	nanotubes	bubble recoil [102]
	magnetic	chain	magnetic forces/torques [103]
	chemical	Janus	bubble recoil [74]
	chemical	Janus	self-diffffusiophoresis [39,104]
	ultrasound	nanoparticle	asymmetric distribution of encapsulated magnetic nanoparticles [105]
	light	Janus	diffffusiophoresis induced by light [106]
	light	micromotors	surface free energy gradient generated by photoisomerization of molecules [107,108]
	light	LCEs	photoisomerization of molecular motor induced deformation [109,110]
	chemical	Janus	surface tension gradients/bubble recoil [111,112]
Biohybrid Technique	biohybrid	micromotors	enzyme-catalyzed reactions [113]
	biohybrid	micromotors	intact motile cells [114]
Use of Original Materials	light	particles	photoinduced self-diffffusiophoresis [76,90]
	electric	micro-objects	bipolar chemistry-induced asymmetric bubble generation [115]

**Table 2:** Summary of stimulus–response mechanisms and applications of micro/nanorobots.

Stimuli	Composition	Response	Application
temperature	NIPAM/AAM/PEGDA/9mTc	Swelling/shrinking	Tracking, imaging [59]
	NIPAM/MNPs	Swelling/shrinking	Treating Cancer [60]
	NIPAM/PEDGA/MNPs/Fe_3_O_4_	Swelling/shrinking	Conditioning movement [183]
	Mg/Pt-NIPAM	Swelling/shrinking	Drug delivery [61]
	LC	Swelling/shrinking	Temperature sensor [184]
	NIPAM/AAc/NaAlg	Swelling/shrinking	Speed and direction adjustment [185]
light	IP-DIP/LCE	Shape transformation	Motion Modulation [16]
	IP-DIP/LCE	Bending deformation	Particle Capture [18]
	Spirulina platensis/Fe_3_O_4_/TiO_2_	Photocatalysis degradation	Removal of organic contaminants [19]
	chitosan	photocleavage	Drug delivery [20]
	biotin/NH_2_–Fe_3_O_4_/streptavidin	Photocleavage	Cancer treatment [17]
	NIPAM-AAM/PEGDA/ SiO_2_-coatedFe_2_O_3_/GO nanosheet	Swelling/shrinking	Drug delivery [21]
	NIPAM/alginate/MNPs	Swelling/shrinking	Drug delivery [23]
	E-dent 400/PDA/MNPS/lipiodol	Photothermal effect	Drug delivery [24]
	Geltin/PVA/MNPs/PLGA	Photothermal effect	Cancer treatment [27]
ultrasound	PLGA/PFC	Cavitation effect	Drug resistance resulting from the induction of lactic acidosis by tumor tissue [2]
	PEGDA/PETA	Acoustic streaming effect	Effect of various drug release patterns on the therapeutic effectiveness of cancer cells [3]
	E-dent 400/NdFeB	Acoustic streaming effect	Reduced stimulus–response time for rapid drug release [4]
	P(VDF-TrFE)/CFO	Acoustic streaming effect	Neuron-like cell trafficking and cell differentiation [5]
	PEDOT/MnO_2_	Cavitation effect	Dynamic assembly, swarming [6]
	Au–Pt	Acoustic streaming effect	Dynamic assembly, swarming [7]
pH	Mg/Au/EUDRAGITU L100-55 Cy5/Apt/Lip	Consumption of local protons	Stomach acid neutralization, drug release [58]
	chitosan/sodium alginate/Fe_3_O_4_	Dissolved under alkaline conditions	Drug delivery [63]
	PHEMA/PEGDA/Fe_3_O_4_	Swelling/shrinking	Drug delivery [64]
	IPL-780/PDA/Ni/Ti	pH-induced bond hydrolysis	Drug delivery [65]
	CoNi/alginate	Swelling/shrinking	Drug delivery [66]
	PPy/Fe_3_O_4_/Pt	Charge change-affinity regulation–aggregation of estrogen fibers	Removal of estrogenic contaminants from water [62]
	Cy_5_/Apt/Lip	Acid Driven—Specific Targeting	Biosensory imaging (ATP) [67]
	AAc/NIPAM/PVP	Expansion, contraction, torsion	Multi-degree-of-freedom shape transformation [186]
	PEGDA/glycerol/CEA	Distortion by swelling	Shape shift [174]
	EMK/AAc/NIPAM/DPEPA	Swelling/shrinking	Shape shift [170]
	EMK/AAc/NIPAM/DPEPA	Module Assembly	Vehicle–human shape shifting [173]
magnetic	GelMA/CFO/BFO	Magnetoelectric effect	Inducing neuron-like cell differentiation [9]
	NdFeB/silicone	Magnetic control	Instant shape locking while moving without constraints [10]
	MnFe_2_O_4_/oleic acid	Hydrophobic interactions—tight magnetic shell layer	Bubble jet to remove oil droplets [12]
	IP-Dip/Ni/Au	Paramagnetic effect	Direction of Motion Adjustment [8]
Biological	gelatin methacryloyl/ poly(ethylene glycol) amine/Fe_3_O_4_	Bond hydrolysis-swelling	Drug delivery [29]
	GelMA/Fe@ZIF-8	Bond hydrolysis	Drug delivery [30]
	GelMA/PEGDA/Fe_3_O_4_	Bond hydrolysis	Cell culture [31]
	gelatin/Fe_3_O_4_/neutrophil	Chemotaxis	Crossing the blood–brain barrier to release drugs [32]
	*E. coli* bacteria	Specific binding	Early Cancer Diagnosis [33]
	DNA	Controlled conformational changes	Early Cancer Diagnosis [34]
ion	alginate/chitosan/Fe_3_O_4_	Ion exchange	Drug delivery [57]
	NIPAM/BC18A6m/MNPs	Chelation sensing Pb^2+^ ion	Heavy metal detection (Pb^2+^) [68]
	ZnO/SiO_2_	Continuous corrosion by H^+^	Detection of CO_2_ [69]

**Table 3:** Challenges and strategies for microrobot applications.

Challenges	Strategies
Synthetic steps (complicated)	laser-based 3D printing
Sensitivity (poor/irreversible response to stimuli)	optimization of structure Design, exploration of sensing principle
Mechanical properties (insufficient mechanical properties to fully support flexible deformation)	increasing the degree of crosslinking, tailoring the hybrid formulation
Biosafety (poor biosafety)	making the most of reported biosafe materials, integrating natural/ physiologically relevant mechanisms into developing biosafe materials
Limited load capacity (individually too small)	development of group drive and control strategies
Visualization (difficult to track)	multimodal joint imaging
Biodegradability (postoperative degradation)	customized recyclable strategies
